# All-cause and immune checkpoint inhibitor–associated acute kidney injury in immune checkpoint inhibitor users: a meta-analysis of occurrence rate, risk factors and mortality

**DOI:** 10.1093/ckj/sfad292

**Published:** 2023-11-28

**Authors:** Jia-Jin Chen, Tao-Han Lee, George Kuo, Chieh-Li Yen, Cheng-Chia Lee, Chih-Hsiang Chang, Kun-Hua Tu, Yung-Chang Chen, Ji-Tseng Fang, Cheng-Chieh Hung, Chih-Wei Yang, Wen-Chi Chou, Ching-Chi Chi, Yu-Kang Tu, Huang- Yu Yang

**Affiliations:** Kidney Research Center, Nephrology Department, Chang Gung Memorial Hospital in Linkou, Chang Gung University College of Medicine, Taoyuan, Taiwan; Nephrology Department, Chansn Hospital, Taoyuan City, Taiwan; Kidney Research Center, Nephrology Department, Chang Gung Memorial Hospital in Linkou, Chang Gung University College of Medicine, Taoyuan, Taiwan; Kidney Research Center, Nephrology Department, Chang Gung Memorial Hospital in Linkou, Chang Gung University College of Medicine, Taoyuan, Taiwan; Kidney Research Center, Nephrology Department, Chang Gung Memorial Hospital in Linkou, Chang Gung University College of Medicine, Taoyuan, Taiwan; Kidney Research Center, Nephrology Department, Chang Gung Memorial Hospital in Linkou, Chang Gung University College of Medicine, Taoyuan, Taiwan; Kidney Research Center, Nephrology Department, Chang Gung Memorial Hospital in Linkou, Chang Gung University College of Medicine, Taoyuan, Taiwan; Kidney Research Center, Nephrology Department, Chang Gung Memorial Hospital in Linkou, Chang Gung University College of Medicine, Taoyuan, Taiwan; Kidney Research Center, Nephrology Department, Chang Gung Memorial Hospital in Linkou, Chang Gung University College of Medicine, Taoyuan, Taiwan; Kidney Research Center, Nephrology Department, Chang Gung Memorial Hospital in Linkou, Chang Gung University College of Medicine, Taoyuan, Taiwan; Kidney Research Center, Nephrology Department, Chang Gung Memorial Hospital in Linkou, Chang Gung University College of Medicine, Taoyuan, Taiwan; Department of Hematology and Oncology, Chang Gung Memorial Hospital in Linkou and College of Medicine, Chang Gung University, Taoyuan, Taiwan; School of Medicine, College of Medicine, Chang Gung University; Department of Dermatology, Chang Gung Memorial Hospital, Linkou, Taoyuan, Taiwan; Graduate Institute of Epidemiology and Preventive Medicine, College of Public Health, National Taiwan University, Taipei, Taiwan; Kidney Research Center, Nephrology Department, Chang Gung Memorial Hospital in Linkou, Chang Gung University College of Medicine, Taoyuan, Taiwan; Department of Health Policy and Management, Johns Hopkins Bloomberg School of Public Health, Baltimore, MD, USA

**Keywords:** acute kidney injury, immune checkpoint inhibitors, immune-related adverse events, mortality, proton pump inhibitors

## Abstract

**Background:**

Immune checkpoint inhibitors (ICIs) have been associated with acute kidney injury (AKI). However, the occurrence rate of ICI-related AKI has not been systematically examined. Additionally, exposure to proton pump inhibitors (PPIs) and non-steroidal anti-inflammatory drugs (NSAIDs) were considered as risk factors for AKI, but with inconclusive results in ICI-related AKI. Our aim was to analyse the occurrence rate of all-cause AKI and ICI-related AKI and the occurrence rates of severe AKI and dialysis-requiring AKI, and to determine whether exposure to PPIs and NSAIDs poses a risk for all-cause and ICI-related AKI.

**Methods:**

This study population was adult ICI recipients. A systematic review was conducted by searching MEDLINE, Embase and PubMed through October 2023. We included prospective trials and observational studies that reported any of the following outcomes: the occurrence rate of all-cause or ICI-related AKI, the relationship between PPI or NSAID exposure and AKI development or the mortality rate in the AKI or non-AKI group. Proportional meta-analysis and pairwise meta-analysis were performed. The evidence certainty was assessed using the Grading of Recommendations Assessment, Development and Evaluation framework.

**Results:**

A total of 120 studies comprising 46 417 patients were included. The occurrence rates of all-cause AKI were 7.4% (14.6% from retrospective studies and 1.2% from prospective clinical trials). The occurrence rate of ICI-related AKI was 3.2%. The use of PPIs was associated with an odds ratio (OR) of 1.77 [95% confidence interval (CI) 1.43–2.18] for all-cause AKI and an OR of 2.42 (95% CI 1.96–2.97) for ICI-related AKI. The use of NSAIDs was associated with an OR of 1.77 (95% CI 1.10–2.83) for all-cause AKI and an OR of 2.57 (95% CI 1.68–3.93) for ICI-related AKI.

**Conclusions:**

Our analysis revealed that approximately 1 in 13 adult ICI recipients may experience all-cause AKI, while 1 in 33 adult ICI recipients may experience ICI-related AKI. Exposure to PPIs and NSAIDs was associated with an increased OR risk for AKI in the current meta-analysis.

## Graphical Abstract

**Graphical Abstract:**
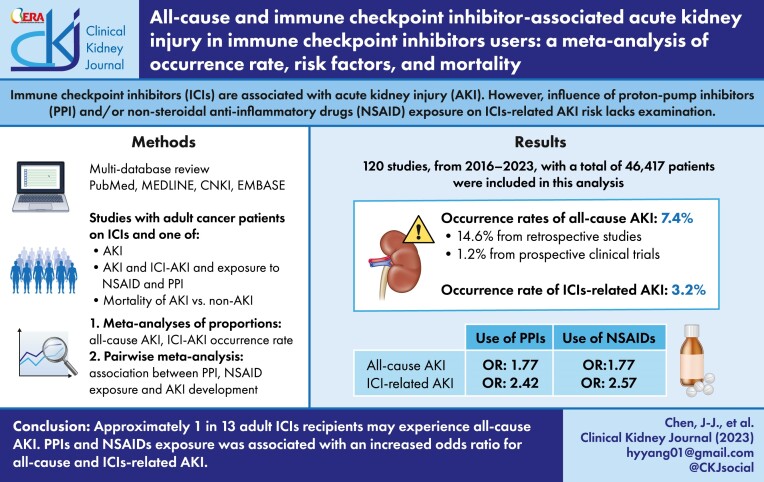

KEY LEARNING POINTS **What was known:**

- The use of immune checkpoint inhibitors (ICIs) might entail a risk for both all-cause AKI and ICI-related AKI. Yet, the occurrence rates of all-cause AKI or ICI-related AKI and the influence of non-steroidal anti-inflammatory drug (NSAID)/proton pump inhibitor (PPI) exposure on ICI-related AKI risk lack systematic examination.

**This study adds:**

- Approximately 1 in 13 and 1 in 33 adult ICI recipients may experience all-cause AKI and ICI-related AKI, respectively. The occurrence rates for severe all-cause AKI and severe ICI-related AKI were 1.8% and 1.2%, respectively. PPI or NSAID exposure was associated with an increased odds ratio for both all-cause and ICI-related AKI in the current study.

**Potential impact:**

- Identifying all-cause and ICI-related AKI is crucial. Further prospective studies with histopathology examination are needed to further explore the true incidence. It is advisable for adult ICI recipients to avoid unnecessary PPI or NSAID exposure.

## INTRODUCTION

Over the past 2 decades, immune checkpoint inhibitors (ICIs) have seen increasing utilization in the treatment of

advanced-stage malignancies, following their initial approval for metastatic melanoma [1, 2]. Three major types of ICIs are focused on different pathways. These include anti-PD-1 antibodies (such as nivolumab, pembrolizumab and cemiplimab), used for the treatment of non-small-cell lung cancer, head and neck cancer, renal cell carcinoma, lymphoma and colon cancer. Additionally, anti-CTLA-4 antibodies (ipilimumab) are employed in the treatment of colon cancer, renal cell carcinoma and melanoma. Lastly, anti-PD-L1 antibodies (such as atezolizumab, avelumab and durvalumab) are utilized for non-small-cell lung cancer, bladder cancer and breast cancer [3]. However, the enhancement of anti-cancer immune responses through the inhibition of negative immunologic regulation pathways has resulted in unique systemic side effects known as immune-related adverse events (irAEs). Renal irAEs have become a topic of interest among nephrologists and oncologists due to the growing prescription of ICIs in recent years [3, 4]. The primary pathological finding in ICI-related acute kidney injury (AKI) is acute tubulointerstitial nephritis (ATIN), although other conditions such as glomerulonephritis or thrombotic microangiopathy have also been reported [3, 4].

Previous publications have demonstrated that the occurrence rate of all-cause AKI in cancer patients treated with ICIs is relatively low in prospective clinical studies [5, 6], compared with a higher rate reported in real-world evidence [7]. When compared with traditional standard therapy, the combination of PD-1 inhibitors or PD-L1 inhibitors with chemotherapy appears to pose a higher risk for all-cause AKI in ICI recipients [8]. Additionally, the use of proton pump inhibitors (PPIs) and non-steroidal anti-inflammatory drugs (NSAIDs) may be associated with an increased risk of all-cause AKI or tubulointerstitial nephritis in the general population [7]. However, published meta-analyses have primarily concentrated on discussions related to all-cause AKI, leaving ICI-related AKI relatively unexplored in a systematic manner. This gap in research can be attributed to the lack of a definitive definition for ICI-related AKI. Furthermore, the intricate mechanisms underlying AKI present a challenge in differentiating ICI-related AKI from AKI caused by other disease-associated factors. Furthermore, the impact of PPI and NSAID exposure on both all-cause AKI and ICI-related AKI in cancer patients treated with ICIs remains inconclusive. Additionally, the occurrence rates of severe AKI and dialysis-requiring AKI have not been studied.

The primary objective of this study was to separately analyse the occurrence rates of all-cause AKI and ICI-related AKI in adult ICI recipients, to assess the occurrence rates of severe AKI and dialysis-requiring AKI.

## MATERIALS AND METHODS

### Literature search strategy

This meta-analysis was performed in accordance with the Preferred Reporting Items for Systematic Reviews and Meta-Analyses guidelines [9]. The protocol was registered with PROSPERO (CRD42022335237). Two independent reviewers (J.J.C. and T.H.L.) conducted a comprehensive systematic review and searched for articles published until 10 October 2023 in PubMed, MEDLINE, China National Knowledge Infrastructure (CNKI) and Embase. Detailed search strategies are provided in Supplementary Table S1. Review articles were not included in the present analysis, however, the references were screened for relevant studies. There were no limitations on language or article type.

### Study eligibility criteria

The titles and abstracts of the studies extracted from the search were independently examined by two reviewers (J.J.C. and T.H.L.) and articles were excluded during initial screening if the titles or abstracts indicated that they were clearly irrelevant to the objective of the current study. The full texts of the relevant articles were reviewed to determine whether the studies were eligible for inclusion.

Studies that enrolled adult patients with malignancies receiving ICIs were included. The other inclusion criteria were that the study reported at least one of the following outcomes of interest: the occurrence rate of AKI with various definitions [either guideline-based AKI criteria such as the Acute Kidney Injury Network (AKIN), Kidney Disease: Improving Global Outcomes (KDIGO), Common Terminology Criteria for Adverse Events (CTCAE) or predefined AKI criteria by individual studies] or ICI-related AKI; all-cause AKI and ICI-related AKI with a record of exposure to PPIs or NSAIDs; and the mortality rates of all-cause AKI or ICI-related AKI versus a non-AKI group.

A third reviewer (G.K.) was consulted in order to reach an agreement through consensus in case of any disagreement regarding eligibility. Studies were excluded if they were duplicate cohorts or had insufficient information about the outcomes.

### Data extraction and outcome measurement

Two investigators (J.J.C. and T.H.L.) independently extracted the outcomes of interest and characteristics information of the included studies. The outcomes of interest were the occurrence rates of all-cause AKI and ICI-related AKI, the occurrence rate of severe AKI and dialysis-requiring AKI, the association between potential risk factors (exposure to PPIs and NSAIDs) and the development of all-cause or ICI-related AKI. Severe AKI in this study was defined based on stage 2–3 AKI according to the KDIGO and AKIN criteria, or as at least grade 3 according to the CTCAE criteria. For studies that reported elevated serum creatinine levels according to CTCAE criteria (staging determined by either the upper normal limit or baseline creatinine level) and did not separately report the number of patients whose stages were determined by baseline creatinine, we considered these studies as having no relevant outcomes. The secondary outcomes aimed to examine the association between the occurrence of AKI in the context of ICI treatment and death.

The present study also extracted relevant variables including the mean age, sex, most common and second most common types of malignancy, AKI definition, ICI-related AKI definition, classification of ICIs and the locations and countries in which the study was performed. Disagreements about data extraction between the two authors (J.J.C. and T.H.L.) were resolved through discussion with a third author (G.K.).

### Data synthesis and analysis

To pool the all-cause AKI occurrence rate and the ICI-related AKI occurrence rate, we employed a random effects model with the inverse variance method in meta-analyses of proportions. We used the restricted maximum likelihood method to estimate between-study variance (τ^2^) and used the Hartung–Knapp procedure to construct confidence intervals (CIs). We conducted subgroup analyses to explore the potential sources of heterogeneity in AKI occurrence rate by dividing the studies according to the study design (retrospective cohort studies or prospective clinical trials), the major malignancy type in each study (melanoma, lung cancer, renal cell carcinoma, urothelial carcinoma, others or not reported), the AKI definition (KDIGO, AKIN, CTACE and other criteria), study location (single centre, multicentre, not reported), mean age (≥65 or <65 years old) and sample size (≤400 or >400).

To examine the relationship between exposure to PPIs or NSAIDs and the development of AKI, and to examine the outcome impact of AKI, the number of patients with exposure, the number of all-cause AKI and ICI-related AKI in the drug exposure and non-exposure groups and the number of mortalities were extracted and pooled odds ratios (ORs) were used to estimate overall effects. In this study we used a random effects model for pooling estimated effects and the Hartung–Knapp method for constructing CIs. The restricted maximum likelihood method was applied to assess between-study variance. Sensitivity analysis was conducted to examine the relationship between drug exposure and the development of AKI, utilizing methods such as multivariate meta-regression (detailed methodology provided in Supplementary Document 1) in considering drug interaction. In addition, we applied the trim-and-fill method [10, 11] and conducted a limited meta-analysis [12] to account for potential publication bias in the studies included in this analysis, as they were predominantly based on published cohort studies. These approaches were utilized to adjust the results regarding drug exposure and the development of AKI.

The binary outcome analysis was conducted using the metabin function and the pooled occurrence rate was analysed using the metaprop function in the R package meta (version 4.18-2; R Foundation for Statistical Computing, Vienna, Austria) [13]. Subgroup analysis in the pooled AKI occurrence rate was conducted using the update.meta function. Heterogeneity was examined using the *I*^2^ index (*I*^2^ < 25%, 25–50% or >50%, indicating mild, moderate and high heterogeneity, respectively). Small study bias was assessed by the arcsine test [14]. The trim-and-fill method was conducted using the Trimfill function in the meta package and limited meta-analysis by the limitmeta function in the metasens package.

We also accessed the quality of evidence regarding PPI and NSAID exposure and the development of AKI by the Grading of Recommendations Assessment, Development and Evaluation (GRADE) methodology [15]. The certainty of evidence regarding AKI as a prognosticator in ICI recipients was assessed by an adapted GRADE framework [16].

### Risk of bias assessment

For pooled all-cause AKI and ICI-related AKI occurrence rate, the Hoy risk of bias tool was used [17, 18]. This tool consists of 10 items (Supplementary Document 2) that are ranked as 1 (yes) or 0 (no). The summed scores range from 8 to 10, with a score of 6–7 indicating moderate risk of bias and a score <6 indicating a high risk of bias. For the prognostic impact of AKI, the Quality In Prognosis Studies tool was used for risk of bias assessment [19]. For PPI and NSAID exposure, we used the Newcastle–Ottawa scale (NOS), which allocates a maximum of 9 points for three major domains: quality of the selection, comparability and outcome of the study populations [20]. Scores ranging from 7 to 9, from 4 to 6 and <4 indicate low, moderate and high risk of bias, respectively. The quality of the enrolled studies was assessed independently by two authors (J.J.C. and T.H.L.). Disagreements between the two investigators were resolved through consensus with a third author (G.K.).

## RESULTS

### Search results and study characteristics

A flowchart of the literature search is provided in Supplementary Fig. S1. The electronic database search identified 353 potentially eligible studies from PubMed, 972 from Embase, 179 from CNKI, 314 from MEDLINE. After removing duplicate articles, the remaining articles were screened. After screening the titles and abstracts, the full texts of 170 studies were reviewed to assess their eligibility. After excluding 50 studies for various reasons [no outcome of interest (*n* = 35), duplication cohort (*n* = 6), adverse report system cohort without event number (*n* = 2), biopsy cohort (*n* = 7)] (Supplementary Table S2), 120 studies comprising 46 594 patients were included for analysis [21–140].

Detailed characteristics of the enrolled studies are presented in Supplementary Table S3 and summarized in Table 1. The most common study design was a prospective clinical trial (73 of the 120 studies) and 4 were matched case–control in design [25, 28, 30, 92]. The most commonly used all-cause AKI criteria (77 of 120) in the enrolled studies was the CTCAE AKI definition, followed by KDIGO criteria (31 of 120), other predefined criteria in 3 studies and AKIN criteria in 2 studies. Seven of the 120 enrolled studies did not report the AKI definition. It should be noted that the outcome of ICI-related AKI was reported in less than half of the enrolled studies (29 of 120). For ICI-related AKI, 14 of 29 relevant studies were determined by nephrologists or oncologists (Supplementary Tables S3 and S4). Other studies defined ICI-related AKI either following the American Society of Clinical Oncology clinical guidelines [5], using an acute interstitial nephritis prediction model [141] with probability >90%, or by its own predefined criteria.

**Table 1:** Summary characteristics of the enrolled studies.

	
Enrolled studies, *N*	120
Enrolled participants, *N*	46 417
Study design, *n*	
Retrospective cohort study	42
Retrospective matched cohort study	4
Prospective clinical trial	73
Prospective observational study	1
Basic demographics^a^	
Age (years), mean	64.2
Female, %	37.9
Country, *n*	
Multiple countries	33
Single country	87
Location, *n*^b^	
Multicentre	67
Single centre	53
Studies with a single cancer type, *n*/participants, *n*	
Lung cancer	20/4992
Melanoma	8/4071
Urothelial cell carcinoma	13/2290
Renal cell carcinoma	11/1927
Breast cancer	5/554
Lymphoma	4/231
Head and neck squamous cell carcinoma	3/203
Skin squamous cell carcinoma	1/131
Multiple myeloma	3/101
Colorectal cancer	2/107
Anal cancer	1/94
Pancreatic cancer	1/91
Merkel cell carcinoma	1/88
Basal Cell carcinoma	1/84
Gastric cancer	1/28
Hepatocellular carcinoma	1/28
Ovarian cancer	1/26
Cervical cancer	1/16
Studies with mixed cancer types, *n*	
Major type: lung cancer	16
Major type: melanoma	7
Major type: renal cell carcinoma	3
Major type: breast cancer	1
Major type: gastric and oesophageal cancer	1
Major type: lymphoma	1
Major type: mesothelioma	1
Major type: urothelial cell carcinoma	1
Major type: other^c^	2
Major type: unknown primary advanced cancer	2
Cancer type not reported, *n*	7

a Mean values were derived from available studies.

b One study did not report the information regarding study location.

c Other type: one study primarily encompassed a mix of lung, breast and head–neck cases, while another study enrolled individuals with unresectable solid tumours.

### Occurrence rate of all-cause AKI and ICI-related AKI

**Figure 1:**
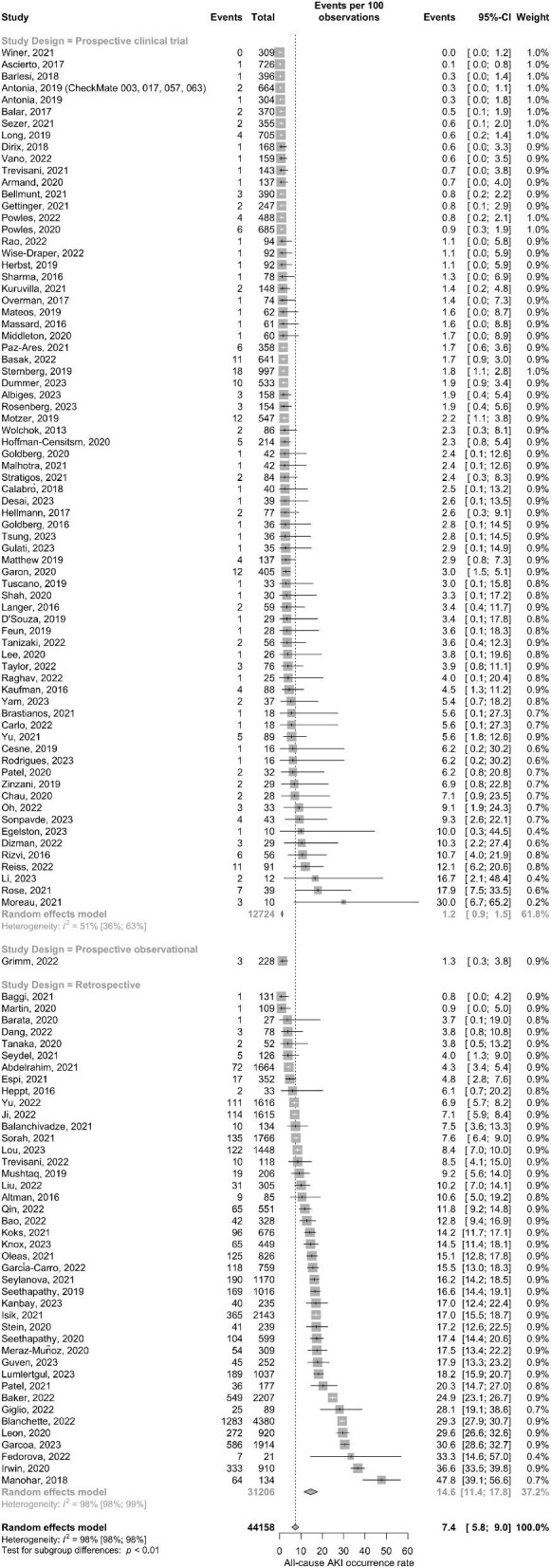
Forest plot of pooled occurrence rate of all-cause AKI.

Three of the four matched case–control studies [25, 28, 30] and one of the retrospective studies reported ICI-related AKI [41] and were therefore excluded. Thus, by including 116 studies with 44 158 patients, the pooled all-cause AKI occurrence rate was 7.4% (95% CI 5.8–9.0) with high heterogeneity [*I*^2^ = 98% (95% CI 98–98)] (Fig. 1). The occurrence rate from retrospective studies was significantly higher than from prospective clinical trials (14.6% versus 1.2%; *P* for subgroup difference <.01).

**Figure 2:**
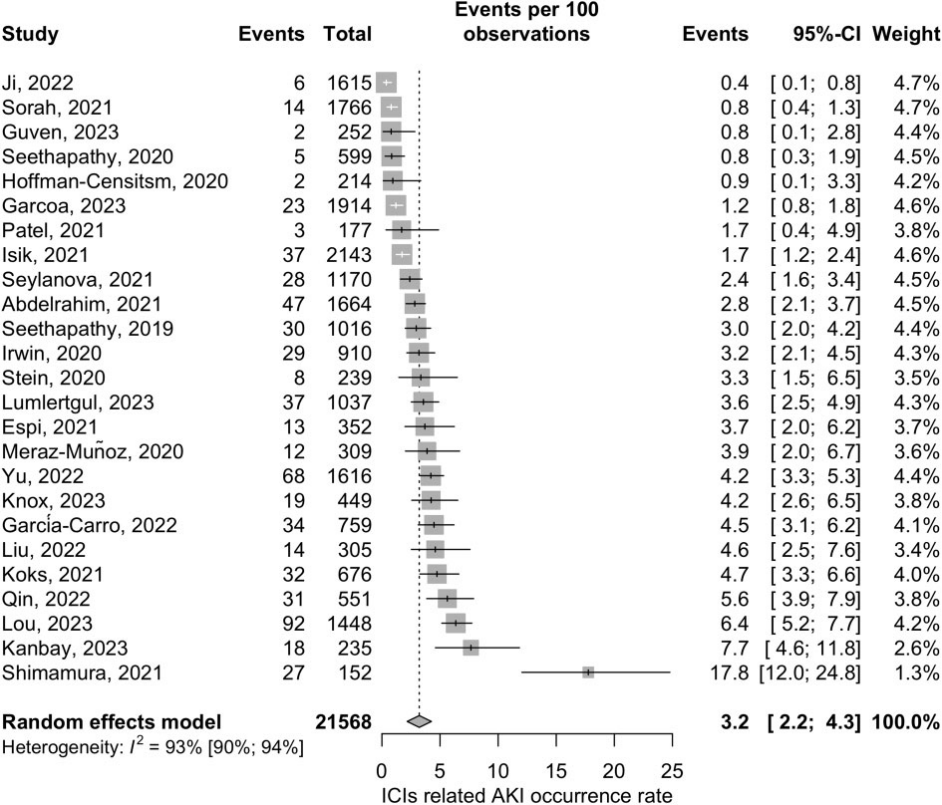
Forest plot of pooled occurrence rate of ICI-related AKI.

By including 25 retrospective cohort studies with 21 568 patients, the pooled ICI-related AKI occurrence rate was 3.2% (95% CI 2.2–4.3) with high heterogeneity [*I*^2^ = 93% (95% CI 90–94)] (Fig. 2). One outlier study [41] was noted.

Subgroup analysis was performed and the enrolled studies were divided into different groups according to AKI definition and major malignancy type. There was a significant subgroup all-cause AKI and ICI-related AKI occurrence rate difference between the studies with different major cancer types and AKI definitions (Supplementary Figs. S2 and S3). Sample size and study location difference also resulted in all-cause AKI occurrence heterogeneity.

### Occurrence rates of severe AKI and dialysis-requiring AKI

**Figure 3:**
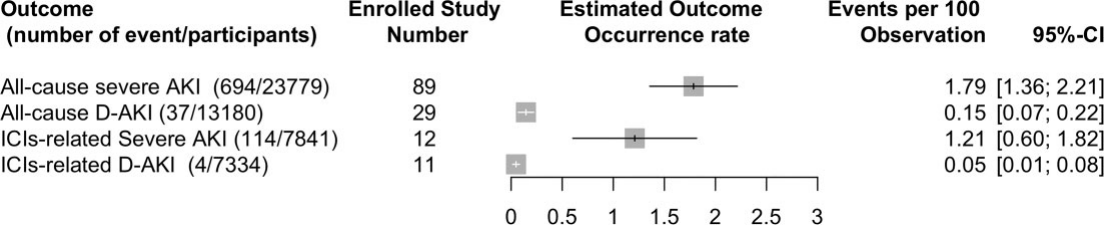
Forest plot of pooled occurrence rate of severe and dialysis-requiring AKI. D-AKI; dialysis requiring acute kidney injury.

The occurrence rates of severe all-cause AKI and severe ICI-related AKI were 1.79% (95% CI 1.36–2.11; pooled occurrence rate: 3.82% from retrospective cohort studies and 0.37% from prospective clinical trials) and 1.21% (95% CI 0.60–1.82), respectively (Fig. 3, Supplementary Figs. S4 and S5). The occurrence rates for dialysis-requiring all-cause AKI and dialysis-requiring ICIs-related AKI were 0.15% (95% CI 0.07–0.22) and 0.05% (95% CI 0.01–0.08), respectively (Fig. 3, Supplementary Figs. S6 and S7).

### Exposure to PPIs and NSAIDs and the development of all-cause AKI and ICI-related AKI

**Figure 4:**
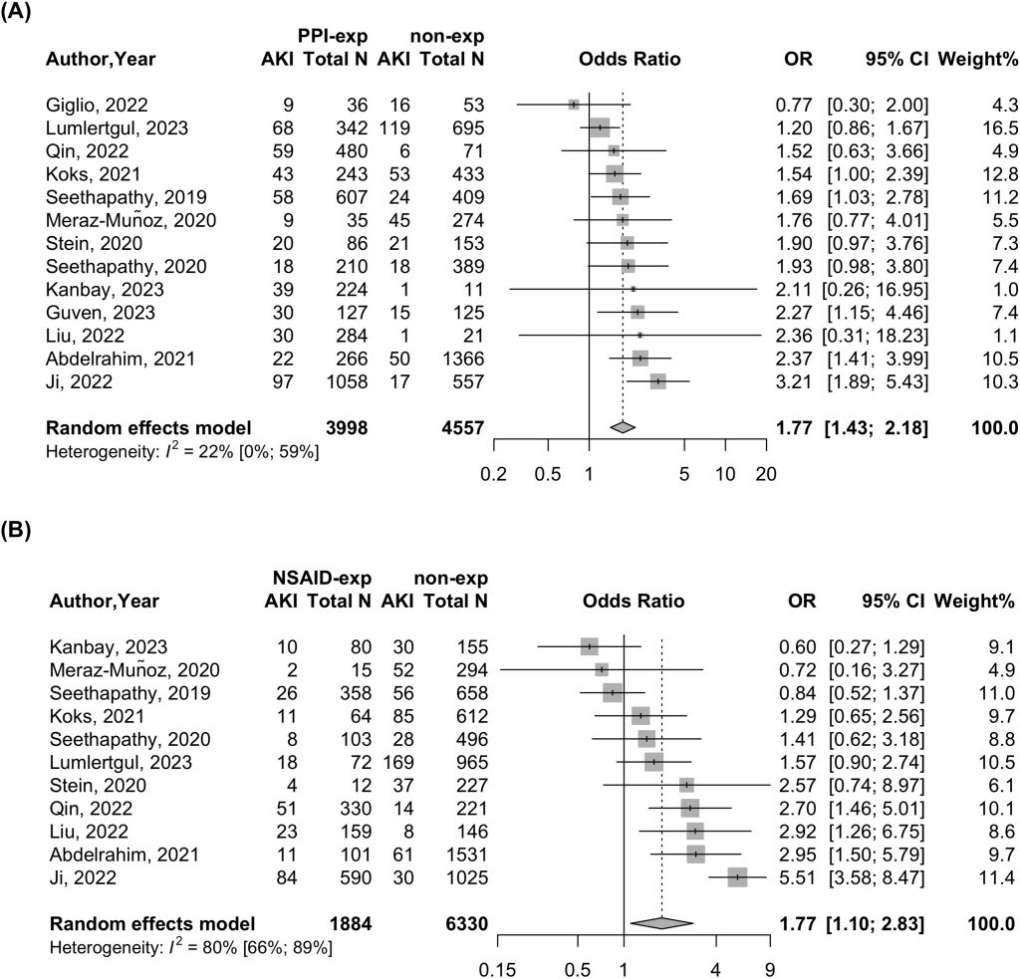
Forest plot of exposure of **(A)** PPIs or **(B)** NSAIDs and the risk of all-cause AKI. The upper middle column shows the AKI event number and total number of participants (total *N*) in the PPI exposure group (PPI-exp) and PPI non-exposure group (non-exp). The lower middle column shows the AKI event number and total number of participants (total *N*) in the NSAID exposure group (NSAID-exp) and NSAID non-exposure group (non-exp).

Thirteen studies with 8555 patients were included to analyse the relationship between PPI exposure and all-cause AKI development. The OR of all-cause AKI was 77% higher for those exposed to PPIs versus those unexposed [OR 1.77 (95% CI 1.43–2.18)] with low heterogeneity [*I*^2^ = 22% (95% CI 0–59)] (Fig. 4A). Eleven studies with 8214 patients were included to analyse the relationship between NSAID exposure and all-cause AKI development. The OR of all-cause AKI was 77% higher for those exposed to NSAIDs versus those unexposed [OR 1.77 (95% CI 1.10–2.83)] with high heterogeneity [*I*^2^ = 80% (95% CI 66–89)] (Fig. 4B).

**Figure 5:**
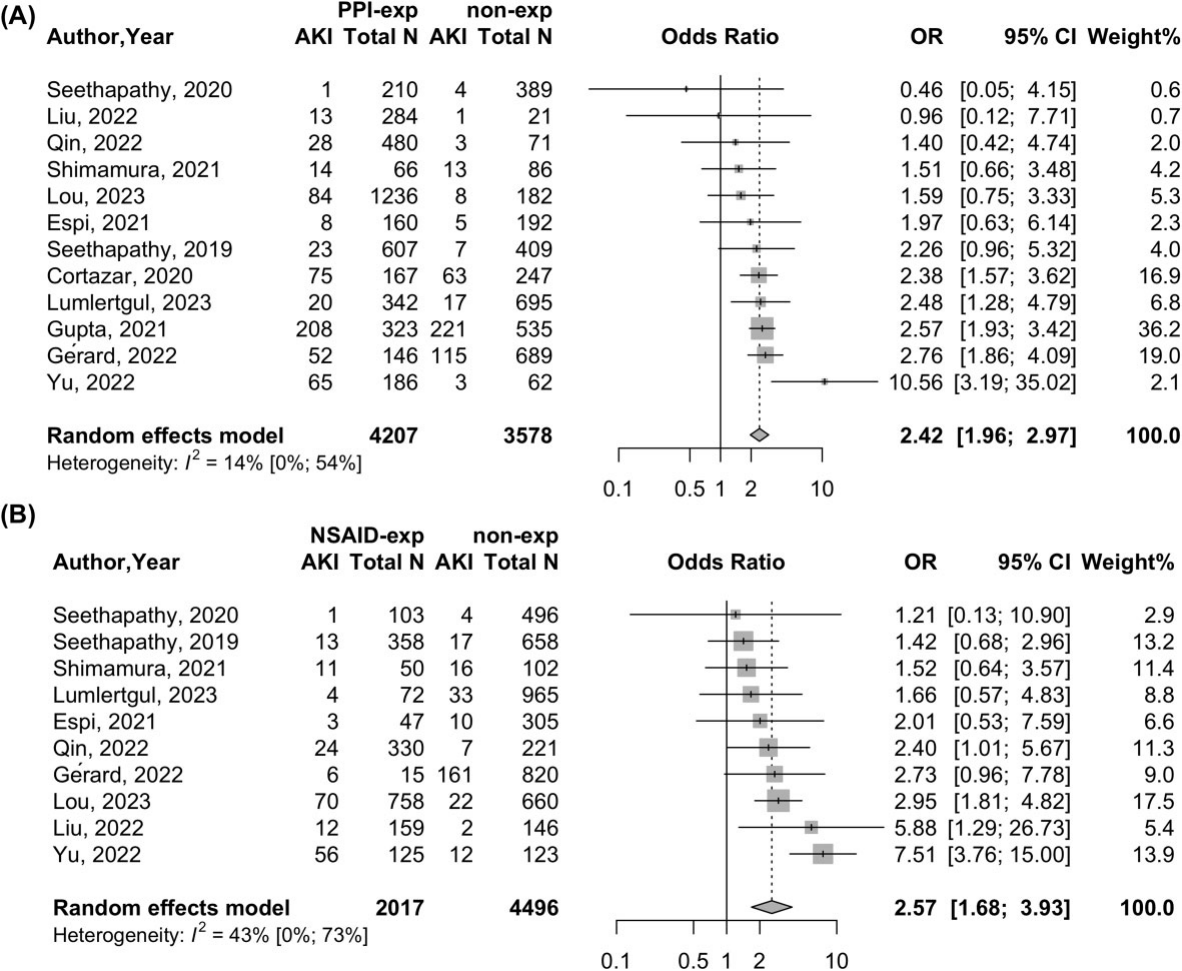
Forest plot of exposure of **(A)** PPIs or **(B)** NSIADs and the risk of ICI-related AKI. The upper middle column shows the AKI event number and total number of participants (total *N*) in the PPI exposure group (PPI-exp) and PPI non-exposure group (non-exp). The lower middle column shows the AKI event number and total number of participants (total *N*) in the NSAID exposure group (NSAID-exp) and NSAID non-exposure group (non-exp).

The OR of ICI-related AKI was 142% higher for those exposed to PPIs versus those unexposed [OR 2.42 (95% CI 1.96–2.97)] with low heterogeneity [*I*^2^ = 14% (95% CI 0–54)] (Fig. 5A). The OR of ICI-related AKI was 157% higher for those exposed to NSAIDs versus those unexposed [OR 2.57 (95% CI 1.68–3.93)] with moderate heterogeneity [*I*^2^ = 43% (95% CI 0–73)] (Fig. 5B).

### Sensitivity analysis

Multivariate meta-regression analysis was conducted to assess the relationship between the use of PPIs or NSAIDs and the development of all-cause AKI and ICIs-related AKI, while accounting for potential correlations within the two drug exposure groups (Supplementary Document 1). The results of the multivariate meta-regression analysis indicated that PPIs and NSAIDs were associated with increased odds of AKI compared with the non-exposure group (Supplementary Table S5). Moreover, the trim-and-fill method and limited meta-analysis further supported these findings, demonstrating that exposure to PPIs or NSAIDs was associated with an increased adjusted OR for both all-cause and ICI-related AKI development when considering potential publication bias (Supplementary Table S5).

### AKI and mortality

In eight studies with 6435 patients, development of AKI had an OR of 1.77 (95% CI 1.06–2.94) for mortality in adult ICI recipients with a heterogeneity of *I*^2^ = 73% (95% CI 45–87) compared with non-AKI patients (Supplementary Fig. S8).

### Risk of bias in enrolled studies

We assessed funnel plot asymmetry using the arcsine test for PPIs as a risk factor for all-cause AKI and ICI-related AKI, obtaining *P*-values of .49 and .23, respectively. For NSAIDs, the *P*-values were .38 for all-cause AKI and .55 for ICI-related AKI (Supplementary Fig. S9). Reporting bias may still exist in the enrolled retrospective cohort studies even though the result of the asymmetry examination was not statistically significant and we conducted further trim-and-fill and limited meta-analysis to examine the robustness of this result (Supplementary Table S5).

The risk of bias was assessed within the 116 studies included in the analysis of all-cause AKI. Among these studies, 43 (37.1%) were classified as having a low risk of bias, while 70 and 3 were categorized as having a moderate and high risk of bias, respectively (Supplementary Table S6). For pooled ICI-related AKI, all studies had moderate risk except one, [73] which was ranked as a high risk of bias (Supplementary Table S7). In the analysis of PPI/NSAID exposure and all-cause AKI development, 12 of 13 studies had a low risk of bias (Supplementary Table S8). Similarly, 7 of 12 studies analysing PPI/NSAID exposure and ICI-related AKI development had a low risk of bias (Supplementary Table S9). The risk of bias within eight studies analysing mortality in ICIs recipients with AKI was also provided (Supplementary Table S10).

### Quality of evidence assessment

The certainty of the evidence of the relationship between PPI and NSAID exposure and AKI development was assessed and is presented in Supplementary Tables S11 and S12. The overall certainty of evidence was low to very low, owing to the nature of observational studies, and the risk of bias was detected in the study limitation domain. The studies included in our analysis were directly relevant to our research question. Therefore, we believe there is no risk associated with the indirectness domain. When examining the relationship between PPI and NSAID exposure and AKI development, we analysed data from >2000 participants. Consequently, we considered the risk of imprecision to be low. The studies examining the association between NSAID exposure and all-cause AKI exhibited high heterogeneity, raising concerns about inconsistency. The certainty of the evidence assessment of the relationship between AKI development and mortality is provided in Supplementary Table S13.

## DISCUSSION

This study revealed four key findings: the occurrence rate of all-cause AKI in real-world studies is 14.6%; the occurrence rate of ICI-related AKI is 3.2%; severe all-cause AKI occurs at a rate of 1.8%, while severe ICI-related AKI occurs at a rate of 1.2%; and PPI or NSAID exposure is associated with an increased OR of both all-cause AKI and ICI-related AKI.

According to the findings of this meta-analysis, the overall pooled occurrence rate of all-cause AKI was 7.4%. Interestingly, a higher occurrence rate of 14.6% was observed when considering real-world evidence. Large cohort studies reported the occurrence rate of AKI in patients with malignancy ranged from 9.3 to 20.2% [142–144]. Carlos *et al.* [145] mentioned this relatively higher rate of AKI occurrence in real-world data than in controlled studies. This discrepancy may be explained by the inclusion of a more complex and critically ill patient population, as well as a higher frequency of exposure to potential nephrotoxic agents in real-world settings. The CTCAE AKI definition might also underestimate the occurrence of mild AKI. Overall, the occurrence rate of AKI in ICI recipients varies across studies, likely due to heterogeneity in patient populations.

Animal models have demonstrated a protective role of PD-L1 in AKI [146] and nephrotoxic agent exposure [147]. One study demonstrated an enrichment of T cells originating from kidney-infiltrating T cells in the urine of ICI-related nephritis patients [148]. The possible mechanism of an increased risk of ICI-related AKI with exposure to nephrotoxic agent is that ICI therapy might disrupt the established immune tolerance of T cells primed toward PPIs [4, 145]. Although a limited number of studies have investigated the relationship between drug exposure and AKI development compared with those examining AKI in ICI recipients, reporting bias should be considered. Despite conducting sensitivity analyses accounting for drug interactions and publication bias, the findings consistently suggest that PPIs and NSAIDs are associated with an increased OR for AKI. However, caution is advised when interpreting these results.

The present study has several strengths. First, we conducted an updated systematic review and meta-analysis to assess the occurrence rate of all-cause AKI and may be the first to evaluate the occurrence rate of ICI-related AKI. Second, we investigated the relationship between PPI and NSAID exposure and the risk of both all-cause AKI and ICI-related AKI development. Lastly, we conducted sensitivity analyses to further examine the association between drug exposure and AKI development.

The present study has several limitations. First, our analysis was conducted using published aggregate data rather than individual patient-level data, which could potentially provide more informative insights. Specifically, for small-scale studies, particularly trials, with limited event occurrences and participant numbers, accurately estimating the true occurrence rate can be challenging. Moreover, while we identified study design, primary cancer type and sample size as potential heterogeneity sources, the lack of detailed baseline data and traditional AKI risk factors, like proteinuria and diabetes mellitus, underscores that significant heterogeneity, not addressed in our analysis, remains a primary limitation. Second, it is important to note the absence of universally accepted criteria for ICI-related AKI. The answer of whether to perform a biopsy in patients with suspected ICI-related AKI remains unclear and may be impractical in routine clinical care. Additionally, further large-scale prospective cohorts may be necessary to comprehensively assess the incidence rate and histopathological features of this condition. The assessment of whether the development of AKI is associated with a long-term risk of chronic kidney disease or end-stage renal disease in adult recipients of ICIs is important; however, this aspect could not be evaluated in the current study. Third, the exposure to PPIs and NSAIDs relied on retrospective cohort or health record studies, with uncertainty regarding concurrent or prior usage. Fourth, we only included published studies in our analysis, which may result in our search for clinical trials being less comprehensive. The aggregated occurrence rate of dialysis-requiring AKI is calculated based on rare events, and numerous studies have not differentiated this outcome from severe AKI. Furthermore, in frail cancer patients, factors such as mortality and the initiation of palliative care could obfuscate accurate estimation of the incidence of dialysis-requiring AKI in this demographic. The aforementioned dilemma extends to analysis of the association between AKI development and mortality, wherein the influence of other underlying disease factors may overshadow the impact of AKI.

## CONCLUSION

In conclusion, our study found a 14.6% occurrence rate of all-cause AKI in real-world cases, with approximately 1 in 33 adult ICI recipients at risk for ICI-related AKI. Further research is needed to explore the role of PPIs and NSAIDs as risk factors and understand their underlying mechanisms. It is advisable to limit unnecessary use of these medications. Large prospective studies, including renal pathology assessments, are warranted to determine the true incidence and histopathological features of ICI-related AKI.

## Supplementary Material

sfad292_Supplemental_File

## Data Availability

The data underlying this article will be shared upon reasonable request to the corresponding author.
