# Supplementary material for: All-cause and immune checkpoint inhibitor–associated acute kidney injury in immune checkpoint inhibitor users: a meta-analysis of occurrence rate, risk factors and mortality
Source: Clin Kidney J. 2023 Nov 28;17(1):sfad292. doi: 10.1093/ckj/sfad292 (PMC10768773; doi:10.1093/ckj/sfad292)
Supplement: sfad292_Supplemental_File [file sfad292_supplemental_file.pdf]

Supplemental Material

Supplemental Table Legends

Supplemental Table 1. Details of search strategy results for each database

Supplemental Table 2. Primary reasons for exclusion and characteristics of excluded studies

Supplemental Table 3. Characteristics of the included studies

Supplemental Table 4. Immune checkpoint inhibitors related AKI definition of the included studies

Supplemental Table 5. Sensitivity analysis of PPI or drug exposure and the risk for all-cause or ICIs-related AKI development

Supplemental Table 6. Risk assessment of the included studies for pooled all-cause AKI occurrence rate

Supplemental Table 7. Risk assessment of the included studies for pooled ICIs-related AKI occurrence rate

Supplemental Table 8. Risk assessment by Newcastle-Ottawa scale regarding drug exposure and all-cause AKI development

Supplemental Table 9. Risk assessment by Newcastle-Ottawa scale regarding drug exposure and ICIs-related AKI development

Supplemental Table 10. Risk assessment of the included cohort studies for AKI and mortality

Supplemental Table 11. GRADE table summarizing the quality of the evidence for PPIs exposure and AKI development

Supplemental Table 12. GRADE table summarizing the quality of the evidence for NSAID exposure and AKI development

Supplemental Table 13. GRADE table summarizing the quality of the evidence regarding AKI as prognostic factor

Supplemental Figure legend

Supplemental Figure 1. PRISMA Flow Diagram

Supplemental Figure 2. Subgroup analysis of pooled occurrence rate of all-cause AKI

Supplemental Figure 3. Subgroup analysis of pooled occurrence rate of ICI-related AKI

Supplemental Figure 4. Pooled occurrence rate of severe all-cause AKI

Supplemental Figure 5. Pooled occurrence rate of severe ICIs-related AKI

Supplemental Figure 6. Pooled occurrence rate of dialysis-requiring all-cause AKI

Supplemental Figure 7. Pooled occurrence rate of dialysis-requiring ICIs-related AKI

Supplemental Figure 8. Forest plot of AKI and the risk of mortality

Supplemental Figure 9. Funnel plot for PPI or NSAID exposure and development of all-cause or ICIs-related AKI

Supplemental Document

Supplemental Document 1. Detailed method and result of multivariate meta-regression

Supplemental Document 2. Risk of Bias Tool

Supplemental Reference

Supplemental Table 1. Details of search strategy results for each database

**Medline through Oct 12st, 2023**	
1#	tubulointerstitial nephritis.mp. or Nephritis, Interstitial/ (6900)
2#	creatinine.mp. or *Creatinine/ (155366)
3#	acute kidney injury.mp. or Acute Kidney Injury/ (71251)
4#	Acute Kidney Injury/ or Nephritis, Interstitial/ or immune-related kidney adverse events.mp. (60183)
5#	1 or 2 or 3 or 4 (216645)
6#	immune checkpoint inhibitor.mp. or Immune Checkpoint Inhibitors/ (14727)
7#	check point inhibitor.mp. (192)
8#	Anti Cytotoxic T-lymphocyte-associated protein 4.mp. (175)
9#	anti CTLA-4.mp. (2059)
10#	Anti PD-1.mp. (8024)
11#	anti programmed death-1.mp. (722)
12#	anti Programmed Cell Death Ligand 1.mp. (187)
13#	anti PD-L1.mp. (3019)
14#	nivolumab.mp. or Nivolumab/ (9666)
15#	pembrolizumab.mp. (8964)
16#	atezolizumab.mp. (3086)
17#	cemiplimab.mp. (371)
18#	ipilimumab.mp. or Ipilimumab/ (5540)
19#	avelumab.mp. (934)
20#	durvalumab.mp. (1518)

21# 6 or 7 or 8 or 9 or 10 or 11 or 12 or 13 or 14 or 15 or 16 or 17 or 18 or 19 or 20 (38012)

22# 5 and 21 (314)

Result: 314

EMbase through Oct 10st, 2023

#1 'adult'/exp AND 'human'/exp AND ('immune checkpoint inhibitor'/exp OR 'immune checkpoint blocker' OR 'immune checkpoint inhibitor' OR 'immune checkpoint inhibitors' OR 'anti cytotoxic t-lymphocyte-associated protein 4' OR 'anti ctla-4' OR 'anti pd-1' OR 'anti programmed death 1 ligand 1'/exp OR 'anti programmed cell death ligand 1' OR 'anti pd-l1' OR 'nivolumab'/exp OR 'pembrolizumab'/exp OR 'atezolizumab'/exp OR 'cemiplimab'/exp OR 'ipilimumab'/exp OR 'avelumab'/exp OR 'durvalumab'/exp) AND ('acute kidney failure'/exp/mj OR 'acute kidney failure' OR 'acute kidney injury' OR 'acute kidney insufficiency' OR 'acute renal failure' OR 'acute renal insufficiency' OR 'kidney acute failure' OR 'kidney failure, acute' OR 'kidney insufficiency, acute' OR 'renal insufficiency, acute' OR 'interstitial nephritis'/exp/mj OR 'acute interstitial nephritis' OR 'interstitial nephritis' OR 'nephritis, interstitial' OR 'tubulointerstitial nephritis' OR 'creatinine'/exp/mj OR 'immune-related kidney adverse events')

Result: 972

CNKI through Oct 11st, 2023

#1 immune checkpoint inhibitor OR immune-related kidney adverse events OR check point inhibitor

#2 acute kidney injury OR tubulointerstitial nephritis (abstract or title)

#3 1 and 2, Filter: Chinese article

Result: 179

Pubmed through Oct 11st, 2023

#1 ((immune checkpoint inhibitor OR check point inhibitor OR Anti Cytotoxic T-lymphocyte-associated protein 4 OR anti CTLA-4 OR Anti PD-1 OR anti programmed death-1 OR anti Programmed Cell Death Ligand 1 OR anti PD-L1) OR (nivolumab[Title/Abstract] OR pembrolizumab[Title/Abstract] OR atezolizumab[Title/Abstract] OR cemiplimab[Title/Abstract] OR ipilimumab[Title/Abstract] OR avelumab[Title/Abstract] OR durvalumab[Title/Abstract])) AND ((((acute kidney injury) OR (tubulointerstitial nephritis)) OR

(creatinine)) OR (immune-related kidney adverse events)) Filters: Adult: 19+ years

Result 751

2 ((immune checkpoint inhibitor OR check point inhibitor OR Anti Cytotoxic T-lymphocyte-associated protein 4 OR anti CTLA-4 OR Anti PD-1 OR anti programmed death-1 OR anti Programmed Cell Death Ligand 1 OR anti PD-L1) OR (nivolumab[Title/Abstract] OR pembrolizumab[Title/Abstract] OR atezolizumab[Title/Abstract] OR cemiplimab[Title/Abstract] OR ipilimumab[Title/Abstract] OR avelumab[Title/Abstract] OR durvalumab[Title/Abstract])) AND ((((acute kidney injury) OR (tubulointerstitial nephritis)) OR (creatinine)) OR (immune-related kidney adverse events)) Filters: Adult: 19+ years

Result: 353

Supplemental Table 2. Primary reasons for exclusion and characteristics of excluded studies

No	Primary Reason for Exclusion	Author, Year	Title	PMID or DOI
1	No outcome of interest	Bajwa, 2019	Adverse Effects of Immune Checkpoint Inhibitors (Programmed Death-1 Inhibitors and Cytotoxic T-Lymphocyte-Associated Protein-4 Inhibitors): Results of a Retrospective Study	30937112
2		Beattie, 2021	Two centres experience of lung cancer resection in patients with advanced non-small cell lung cancer upon treatment with immune checkpoint inhibitors: safety and clinical outcomes	34331065
3		Bernier, 2019	Do immune checkpoint inhibitors increase risk of NSAID and/or PPI renal toxicity?	10.1111:fcp.12470
4		Brahmer, 2015	Nivolumab versus Docetaxel in Advanced Squamous-Cell Non–Small-Cell Lung Cancer	26028407
5		Fuchs, 2018	Safety and Efficacy of Pembrolizumab Monotherapy in Patients With Previously Treated Advanced Gastric and Gastroesophageal Junction Cancer Phase 2 Clinical KEYNOTE-059 Trial	29543932
6		Gadgeel, 2020	Updated Analysis From KEYNOTE-189: Pembrolizumab or Placebo Plus Pemetrexed and Platinum for Previously Untreated Metastatic Nonsquamous Non-Small-Cell Lung Cancer	32150489
7		Gallego, 2022	Incidence and characteristics of adverse drug reactions in a cohort of patients treated with PD-1/PD-L1 inhibitors in real-world practice	36072949
8		Gandhi, 2018	Pembrolizumab plus Chemotherapy in Metastatic Non–Small-Cell Lung Cancer	29658856
9		Cathcart-Rake, 2020	A population-based study of immunotherapy-related toxicities in lung cancer	32446852
10		Chien, 2021	Phase II study of azacitidine with pembrolizumab in patients with intermediate-1 or higher-risk myelodysplastic syndrome	34340254
11		Chowdhury, 2021	A Phase I/II study to assess the safety and efficacy of pazopanib and pembrolizumab combination therapy in patients with advanced renal cell carcinoma	34006498
12		Eskander, 2023	Pembrolizumab Plus Chemotherapy in Advanced Endometrial Cancer	36972022
13		Govindan, 2017	Phase III Trial of Ipilimumab Combined With Paclitaxel and Carboplatin in Advanced Squamous Non–Small-Cell Lung Cancer	28854067
14		Gupta, 2022	Shorter versus longer corticosteroid duration and recurrent immune checkpoint inhibitor-associated AKI	36137651

15		Janjigian, 2020	First-line pembrolizumab and trastuzumab in HER2-positive esophagogastric cancer	32437664
16		Kato, 2021	Concomitant Proton Pump Inhibitors and Immune Checkpoint Inhibitors Increase Nephritis Frequency	34410975
17		Konishi, 2018	Comparison of axitinib and sunitinib as first-line therapies for metastatic renal cell carcinoma: a real-world multicenter analysis	30474747
18		Lacovelli, 2022	First-line avelumab for patients with PD-L1-positive metastatic or locally advanced urothelial cancer who are unfit for cisplatin	35926813
19		Lee, 2021	Lenvatinib in Combination With Pembrolizumab in Patients with Treatment-Naïve and Treatment-Experienced Metastatic Renal Cell Carcinoma: Results From a Phase 1b/2 Study	34143969
20		Long, 2017	Standard-dose pembrolizumab in combination with reduced-dose ipilimumab for patients with advanced melanoma (KEYNOTE-029): an open-label, phase 1b trial	28729151
21		Luangnara, 2022	Incidence and factors associated with cutaneous immune-related adverse events to immune check point inhibitors: An ambispective cohort study	36341419
22		Meyer, 2022	Phase Ib study of BI 836880 (VEGF/Ang2 inhibitor) plus ezabenlimab (BI 754091; anti-PD- 1 antibody) in patients (pts) with advanced hepatocellular carcinoma (HCC).	10.1200:JCO.2022.40.4-suppl.434
23		Maccan, 2022	INCIDENCE AND OUTCOMES OF RENAL ADVERSE EVENTS FOLLOWING TREATMENT WITH IMMUNE CHECKPOINT INHIBITORS	10.1111/nep.14099
24		Moore, 2021	Atezolizumab, Bevacizumab, and Chemotherapy for Newly Diagnosed Stage III or IV Ovarian Cancer: Placebo-Controlled Randomized Phase III Trial (IMagyn050/GOG 3015/ENGOT-OV39)	33891472
25		Nemoto, 2022	Efficacy and safety of immune checkpoint inhibitors in elderly patients with metastatic renal cell carcinoma	34704214
26		Nowakowski, 2022	Safety and efcacy of durvalumab with R-CHOP or R2 -CHOP in untreated, high-risk DLBCL: a phase 2, open-label trial	34797531
27		Patel, 2021	A Phase II Basket Trial of Dual Anti–CTLA–4 and Anti–PD–1 Blockade in Rare Tumors (DART) SWOG S1609: High-Grade Neuroendocrine Neoplasm Cohort	33882143
28		Ravi, 2020	Evaluation of the Safety and Efficacy of Immunotherapy Rechallenge in Patients With Renal Cell Carcinoma	32469396
29		Robert, 2019	Pembrolizumab versus ipilimumab in advanced melanoma (KEYNOTE-006): post-hoc 5-year results from an open-label, multicentre, randomised, controlled, phase 3 study	31345627
30		Sukari, 2019	Cancer Site and Adverse Events Induced by Immune Checkpoint Inhibitors: A Retrospective Analysis of Real-life Experience at a Single Institution	30711957

31		Szabados, 2021	Toxicity and Surgical Complication Rates of Neoadjuvant Atezolizumab in Patients with Muscle-invasive Bladder Cancer Undergoing Radical Cystectomy: Updated Safety Results from the ABACUS Trial	33612455
32		Tawbi, 2017	Pembrolizumab in advanced soft-tissue sarcoma and bone sarcoma (SARC028): a multicentre, two-cohort, single-arm, open-label, phase 2 trial	28988646
33		Tiu, 2023	Safety of Immune Checkpoint Inhibitors in Patients With Advanced Chronic Kidney Disease: A Retrospective Cohort Study	36821637
34		Trevisani, 2021	RENAL FUNCTIONS OUTCOME IN METASTATIC NON SMALL LUNG CARCINOMA PATIENTS: THE RISK OF AKI IN FIRST LINE THERAPY	10.1093:ndt:gfab092.0031
35		Weber, 2016	Safety Profile of Nivolumab Monotherapy: A Pooled Analysis of Patients With Advanced Melanoma	28068177
36	Adverse report system cohort	Hu, 2021	Renal toxicities in immune checkpoint inhibitors with or without chemotherapy: An observational, retrospective, pharmacovigilance study leveraging US FARES database	34845857
37		Qu, 2021	Nephrotoxicity of Immune Checkpoint Inhibitors: A Disproportionality Analysis from 2013 to 2020	34433731
38	Biopsy cohort	Cortazar, 2016	Clinicopathological features of acute kidney injury associated with immune checkpoint inhibitors	27282937
39		Draibe, 2020	Acute tubulointerstitial nephritis induced by checkpoint inhibitors versus classical acute tubulointerstitial nephritis: are they the same disease?	33777371
40		Gérard, 2021	Immune checkpoint inhibitors-induced nephropathy: a French national survey	34155532
41		Giacomo, 2022	Brief Communication PD1-related Nephrotoxicity: Optimizing Its Clinical Management Through Histopathologic Features	35132002
42		Hultin, 2019	Histological diagnosis of immune checkpoint inhibitor induced acute renal injury in patients with metastatic melanoma: a retrospective case series report	32894101
43		Mamlock, 2019	Nephrotoxicity of immune checkpoint inhibitors beyond tubulointerstitial nephritis: single-center experience	30612580
44		Monohar, 2020	Acute Interstitial Nephritis and Checkpoint Inhibitor Therapy	35372854
45	Duplication cohort	Carro, 2021	Nephrotoxicity associated with immune checkpoint inhibitors	10.1111:eci.13567
46		Gu¨ ven, 2021	THE INCIDENCE AND RISK FACTORS FOR ACUTE KIDNEY INJURY IN PATIENTS TREATED WITH IMMUNE CHECKPOINT INHIBITORS: A REAL-LIFE STUDY	10.1093:ndt:gfab082.0022

47		Gupta, 2022	Acute kidney injury in patients receiving pembrolizumab combination therapy versus pembrolizumab monotherapy for advanced lung cancer	35964800
48		Seethapathy, 2021	Immune-related adverse events and kidney function decline in patients with genitourinary cancers treated with immune checkpoint inhibitors	34482189
49		Strohbehn, 2021	Immune checkpoint inhibitor-induced thyroiditis is a risk factor for acute and chronic kidney dysfunction	34383953
50		Su, 2022	接受免疫检查点抑制剂治疗患者发生 急性肾损伤的相关危险因素分析	10.3969/j.issn.1001-9057.2022.01.006

Supplemental Table 3. Characteristics of the included studies

Study	Years of Data Collection	Study design	Country	Location	Mean Age	%, Female	Primary cancer type (%)	ICIs type			All-cause AKI definition	ICIs-related AKI definition	Number Enrolled	Number of all-cause AKI
								CTLA-4 inhibitor	PD-1 inhibitor	PD-L1 inhibitor				
Abdelrahim, 2021	2010 to 2019	Retrospective	U.S.	Single-center	63	34.01	Melanoma: 100%	V	V	V	KDIGO AKI criteria (7 days criteria)	1	1664	72
Albiges, 2023	2021 to 2022	Phase 2 trial	Multi-countries	Multi-center	59.4	29	RCC 100%	X	X	V	CTCAE	Not report ICIs-AKI	158	3
Altman, 2016	NA	Retrospective	U.S.	Single-center	NR	NR	Melanoma 100%	V	V	X	NR	Not report ICIs-AKI	85	9
Antonia, 2019	2013 to 2017	Prospective clinical Trial	Multi-countries	Multi-center	65	43.8	Lung 100%	X	V	X	CTCAE	Not report ICIs-AKI	304	1
Antonia, 2019 (CheckMate 003, 017, 057, 063)	2012 to 2015	Prospective clinical Trial	Multi-countries	Multi-center	63	37	Lung 100%	X	V	X	CTCAE	Not report ICIs-AKI	664	2
Armand, 2020	2014 to 2016	Prospective clinical Trial	U.S	Multi-center	53.6	39.4	classical Hodgkin lymphoma 38.0%	V	V	X	CTCAE	Not report ICIs-AKI	137	1
Ascierto, 2017	2021	Phase 3 trial	Multi-countries	Multi-center	62	38.1	Melanoma 100%	V	X	X	CTCAE	Not report ICIs-AKI	726	1
Baggi, 2021	2019 to 2020	Retrospective	Italy	Multi-center	79	31.3	Cutaneous SCC 100%	X	V	X	CTCAE	Not report ICIs-AKI	131	1
Baker, 2022	2013 to 2019	Retrospective	U.S.	Multi-center	66.5	43.50	Lung 40.14%	V	V	V	KDIGO	2	2207	549
Balanchivadze, 2021	2017 to 2020	Retrospective	U.S	Single-center	66.5	51.5	Lung 100%	X	V	X	CTCAE	Not report ICIs-AKI	134	10
Balar, 2017 (KEYNOTE-052)	2015 to 2016	Prospective clinical Trial	Multi-countries	Multi-center	74	23	UCC 100%	X	V	X	CTCAE	Not report ICIs-AKI	370	2
Barata, 2020	2017 to 2019	Retrospective	U.S	Multi-center	64	30	RCC 100%	X	V	V	CTCAE	Not report ICIs-AKI	27	1
Bao, 2022	2018 to 2021	Retrospective	China	Single-center	63.3	19.5	Lung 100%	X	V	V	25% decrease in eGFR from baseline	Not report ICIs-AKI	328	42
Barlesi, 2018	2015 to 2017	Prospective clinical Trial	Multi-countries	Multi-center	64	32	Lung 100%	X	V	X	CTCAE	Not report ICIs-AKI	396	1
Basak, 2022	2015 to 2020	Prospective clinical Trial	Netherlands	Single-center	66	35	Melanoma 46%	V	V	X	CTCAE	Not report ICIs-AKI	641	11
Bellmunt, 2021 (IMvigor010 trial)	2015 to 2018	Prospective clinical Trial	Multi-countries	Multi-center	67	21	UCC 100%	X	X	V	CTCAE	Not report ICIs-AKI	390	3
Blanchette, 2022	2012 to 2018	Retrospective	Canada	NR	NR	NR	NR	NR	NR	NR	KDIGO	Not report ICIs-AKI	4380	1283

Study	Years of Data Collection	Study design	Country	Location	Mean Age	%, Female	Primary cancer type (%)	ICIs type			All-cause AKI definition	ICIs-related AKI definition	Number Enrolled	Number of all-cause AKI
								CTLA-4 inhibitor	PD-1 inhibitor	PD-L1 inhibitor				
Brastianos, 2021	2018 to 2021	Phase 2 trial	U.S	Multi-center	54	61.1	Breast cancer 44.4%	V	V	X	CTCAE	Not report ICIs-AKI	18	1
Calabrò, 2018	2015 to 2016	Prospective clinical Trial	Italy	Single-center	64	28	mesothelioma 100%	V	X	V	CTCAE	Not report ICIs-AKI	40	1
Carlo, 2022	2016 to 2020	Phase 2 trial	U.S	Single-center	60	38.9	RCC 100%	X	V	X	CTCAE	Not report ICIs-AKI	18	1
Cesne, 2019	2015 to 2017	Prospective clinical Trial	France	Multi-center	41	41.2	Lung 64.7%	X	V	X	CTCAE	Not report ICIs-AKI	16	1
Chau, 2020	2016 to 2017	Prospective clinical Trial	Multi-countries	Multi-center	63	25	Gastric 100%	X	V	X	CTCAE	Not report ICIs-AKI	28	2
Cortazar, 2020	NA	Retrospective (1:2 case control)	Canada and U.S.	Multi-center	65.7	38.60	Lung 34.3%	V	V	V	Sustained an episode of AKI (defined as > 50% increase in SCr)	3	414	138
Dang, 2022	2018 to 2021	Retrospective	France	Single-center	73	26.9	UCC 100%	X	V	X	CTCAE	Not report ICIs-AKI	78	3
Desai, 2023	2017 to 2020	Prospective clinical Trial	Multi-countries	Multi-center	67.9	46.2	unresectable solid tumours	X	X	V	CTCAE	Not report ICIs-AKI	39	1
Dizman, 2022	2019 to 2020	Phase 1 trial	U.S	Single-center	65.3	27.6	RCC 100%	V	V	X	CTCAE	Not report ICIs-AKI	29	3
Dirix, 2018	2013 to 2015	Phase 1 trial	Multi-countries	Multi-center	55	99.4	Breast cancer 100%	X	X	V	CTCAE	Not report ICIs-AKI	168	1
D'Souza, 2019	2015 to 2017	Phase 2 trial	U.S	Multi-center	59	34.5	Multiple myeloma 100%	X	V	X	CTCAE	Not report ICIs-AKI	29	1
Dummer, 2023	2015 to 2017	Phase 3 trial	Multi-countries	Multi-center	59	41	Melanoma 100%	V	V	X	CTCAE	Not report ICIs-AKI	533	10
Egelston, 2023	2016 to 2019	Phase 1 trial	U.S	Multi-center	62	NA	Breast cancer 100%	X	V	X	CTCAE	Not report ICIs-AKI	10	1
Espi, 2021	2015 to 2017	Retrospective	France	Single-center	67	36.60	Melanoma: 53.7%	V	V	V	KDIGO	1	352	17
Fedorova, 2022	2017 to 2020	Retrospective	Russia	Single-center	29	43	Lymphoma 100%	X	V	X	CTCAE	Not report ICIs-AKI	21	7
Feun, 2019	NA	Prospective clinical Trial	U.S	Single-center	67	13.8	Liver 100%	X	V	X	CTCAE	Not report ICIs-AKI	28	1
García-Carro, 2022	2018 to 2019	Retrospective	Spain	Single-center	64	40.80	Lung 31.5%	V	V	V	KDIGO	1	759	118
Garcoa, 2023	2015 to 2020	Retrospective	U.S	Single-center	64.9	43	Lung 34%	NR	NR	NR	serum creatinine to 1.5 times the baseline or an increase of ⩾0.3 mg/dL	4	1914	586
Garon, 2020	2016 to 2017	Prospective clinical Trial	U.S	Multi-center	65	38	Lung 100%	X	X	V	CTCAE	Not report ICIs-AKI	405	12

Study	Years of Data Collection	Study design	Country	Location	Mean Age	%, Female	Primary cancer type (%)	ICIs type			All-cause AKI definition	ICIs-related AKI definition	Number Enrolled	Number of all-cause AKI
								CTLA-4 inhibitor	PD-1 inhibitor	PD-L1 inhibitor				
Gérard, 2022	1985 to 2020	Retrospective (1:4 case control, renal irAE vs non-renal irAE)	France	Multi-center	65.5	38.00	Lung 39.5%	V	V	V	NR	1	835	167
Gettinger, 2021	2018 to 20201	Phase 3 trial	U.S	Multi-center	67.5	33	Lung 100%	V	V	X	CTCAE	Not report ICIs-AKI	247	2
Giglio, 2022	2020 to 2021	Retrospective	Italy	Single-center	69	39.30	Lung 100%	X	V	X	KDIGO	Not report ICIs-AKI	89	25
Goldberg, 2016	2014 to 2015	Prospective clinical Trial	U.S.	Single-center	62	50	Melanoma 50%; non-small cell lung cancer 50%	X	V	X	NA	Not report ICIs-AKI	36	1
Goldberg, 2020	2014 to 2018	Prospective clinical Trial	U.S	Single-center	60	67	Lung 100%	X	V	X	CTCAE	Not report ICIs-AKI	42	1
Grimm, 2022	2016 to 2018	Prospective, obervational	Germany	Multi-center	70	29	RCC 100%	X	V	X	CTCAE	Not report ICIs-AKI	228	3
Gulati, 2023	2018 to 2021	Phase 2 trial	U.S	Single-center	64	32	Head-Neck SqCC	X	X	V	CTCAE	Not report ICIs-AKI	35	1
Gupta, 2021	2012 to 2020	Retrospective (1:1 case control)	Multi-countries	Multi-center	66.5	39.75	Lung 30.2%	V	V	V	NA	3	858	429
Guven, 2023	2014 to 2021	Retrospective	Turkey	Single-center	59	37.7	RCC 22.6%	V	V	V	KDIGO	1	252	45
Hellmann, 2017 (CheckMate 012)	2014 to 2015	Prospective clinical Trial	U.S	Multi-center	65	46.8	Lung 100%	V	V	X	CTCAE	Not report ICIs-AKI	77	2
Heppt, 2016	2015	Retrospective	Germany	Multi-center	72	42.4	Melanoma 100%	V	V	X	NA	Not report ICIs-AKI	33	2
Herbst, 2019	2015 to 2016	Phase 1 trial	Multi-countries	Multi-center	61.4	28.3	Gastric and esophageal cancer 44.6%	X	V	X	CTCAE	Not report ICIs-AKI	92	1
Hoffman-Censitsm, 2020	2015 to 2016	Prospective clinical Trial	U.S	Multi-center	69	24	UCC 100%	X	X	V	CTCAE	4	214	5
Irwin, 2020	2013 to 2019	Retrospective	U.S	Single-center	NA	NA	NA	V	V	X	NA	4	910	333
Isik, 2021	2014 to 2020	Retrospective	U.S.	Single-center	67	50	Lung 42%	V	V	V	AKI >1.5-fold increase in serum creatinine from baseline (Grade 1 kidney toxicity)	3	2143	365

Study	Years of Data Collection	Study design	Country	Location	Mean Age	%, Female	Primary cancer type (%)	ICIs type			All-cause AKI definition	ICIs-related AKI definition	Number Enrolled	Number of all-cause AKI
								CTLA-4 inhibitor	PD-1 inhibitor	PD-L1 inhibitor				
Ji, 2022	2014 to 2019	Retrospective	China	Single-center	57.4	31	Lung 35.2%	V	V	V	KDIGO	4	1615	114
Kanbay, 2023	2016 to 2021	Retrospective	Turkey	Single-center	62.9	42.5	Head-Neck-Lung-Breast 63.4%	X	V	V	KDIGO	1	235	40
Kaufman, 2016	2014 to 2015	Prospective clinical Trial	Multi-countries	Multi-center	72.5	26	Merkel cell carcinoma 100%	X	X	V	NA	Not report ICIs-AKI	88	4
Knox, 2023	2013 to 2021	Retrospective	Australia	Single-center	65	39	Lung 100%	NR	NR	NR	KDIGO	1	449	65
Koks, 2021	2013 to 2020	Retrospective	Netherlands	Single-center	64	37.90	Melanoma: 47.2%	V	V	V	KDIGO	3	676	96
Kuruvilla, 2021	2016 to 2018	Phase 3 trial	Multi-countries	Multi-center	36	44	Lymphoma 100%	X	V	X	CTCAE	Not report ICIs-AKI	148	2
Langer, 2016	2014 to 2016	Prospective clinical Trial	U.S. and Taiwan	Multi-center	62.5	63	Lung 100%	X	V	X	CTCAE	Not report ICIs-AKI	59	2
Lee, 2020	2016 to 2017	Prospective clinical Trial	U.S	Multi-center	59.4	100	Ovarian cancer 100%	X	V	X	CTCAE	Not report ICIs-AKI	26	1
Leon, 2020	2013 to 2019	Retrospective	U.S	Single-center	NR	NR	NR	NR	NR	NR	KDIGO	4	920	272
Li, 2023	2018 to 2020	Phase 1 trial	U.S	Single-center	66.5	41.7	RCC 100%	X	X	V	CTCAE	Not report ICIs-AKI	12	1
Liu, 2022	2018 to 2020	Retrospective	China	Single-center	64	19.4	Lung 100%	X	V	X	KDIGO	1	305	31
Long, 2019 (KEYNOTE-252)	2016 to 2017	Prospective clinical Trial	Multi-countries	Multi-center	63.5	40	Melanoma 100%	X	V	X	CTCAE	Not report ICIs-AKI	705	4
Lou, 2023	2018 to 2022	Retrospective	China	Single-center	66	27.7	Lung 32.6%	X	V	V	KDIGO	4	1448	122
Lumlertgul, 2023	2011 to 2020	Retrospective	U.K.	Single-center	63	40	Lung 36%	V	V	X	KDIGO	1	1037	189
Malhotra, 2021	2017 to 2019	Prospective clinical Trial	Multi-countries	Multi-center	61.5	45.2	Lung 100%	V	V	X	CTCAE	Not report ICIs-AKI	42	1
Manohar, 2018	NR	Retrospective	U.S	Single-center	NR	NR	NR	X	V	X	AKIN	Not report ICIs-AKI	134	64
Martin, 2020	2014 to 2016	Retrospective	Argentima	Multi-center	65	42.2	Lung 100%	X	V	X	CTCAE	Not report ICIs-AKI	109	1
Massard, 2016	2014 to 2015	Prospective clinical Trial	Multi-countries	Multi-center	66	31.1	UCC 100%	X	X	V	CTCAE	Not report ICIs-AKI	61	1

Study	Years of Data Collection	Study design	Country	Location	Mean Age	%, Female	Primary cancer type (%)	ICIs type			All-cause AKI definition	ICIs-related AKI definition	Number Enrolled	Number of all-cause AKI
								CTLA-4 inhibitor	PD-1 inhibitor	PD-L1 inhibitor				
Mateos, 2019	2014 to 2020	Prospective clinical Trial	U.S.	Multi-center	NR	NR	Multiple myeloma 100%	X	V	X	CTCAE	Not report ICIs-AKI	62	1
Matthew 2019	2015 to 2018	Phase 1/2 trial	Multi-countries	Multi-center	65	39	RCC 21.9%	X	V	X	CTCAE	Not report ICIs-AKI	137	4
Meraz-Muñoz, 2020	2010 to 2017	Retrospective	Canada	Single-center	61	39.8	Melanoma: 84.8%	V	V	X	KDIGO	1	309	54
Middleton, 2020	2017 to 2018	Prospective clinical Trial	U.K.	Multi-center	72	45	Lung 100%	X	V	X	NA	Not report ICIs-AKI	60	1
Moreau, 2021	2016 to 2017	Prospective clinical Trial	U.S.	Multi-center	61	40	Multiple myeloma 100%	X	V	X	CTCAE	Not report ICIs-AKI	10	3
Motzer, 2019	2014 to 2016	Phase 3 trial	Multi-countries	Multi-center	NA	NA	RCC 100%	V	V	X	CTCAE	Not report ICIs-AKI	547	12
Mushtaq, 2019	2015 to 2016	Retrospective	U.S.	Single-center	NA	NA	Melanoma 81%	V	V	X	serum creatinine by > 0.3 mg/dL or ≥ 50% from baseline	Not report ICIs-AKI	206	19
Oh, 2022	2018 to 2020	Phase 2 trial	Korea	Single-center	59	18.2	Colorectal cancer 100%	X	X	V	CTCAE	Not report ICIs-AKI	33	3
Oleas, 2021	NR	Retrospective	Spain	Single-center	67	50	Lung 62%	NR	NR	NR	AKIN	Not report ICIs-AKI	826	125
Overman, 2017	2014 to 2016	Phase 2 trial	Multi-countries	Multi-center	52.5	41	Colorectal cancer 100%	X	V	X	CTCAE	Not report ICIs-AKI	74	1
Patel, 2020	2017 to 2018	Phase 2 trial	U.S	Multi-center	60.5	41	Lung 19%	V	V	X	CTCAE	Not report ICIs-AKI	32	2
Patel, 2021	2014 to 2018	Retrospective	U.S.	Single-center	NR	NR	RCC 100%	NR	NR	NR	Cr values demonstrating a ≥ 1.5-fold increase over baseline during ICI therapy	3	177	36
Paz-Ares, 2021	2017 to 2019	Phase 3 trial	Multi-countries	Multi-center	65	30	Lung 100%	V	V	X	CTCAE	Not report ICIs-AKI	358	6
Powles, 2020	2015 to 2017	Prospective clinical Trial	Multi-countries	Multi-center	67.5	26.6	UCC 100%	V	X	V	CTCAE	Not report ICIs-AKI	685	6
Powles, 2022	2017 to 2019	Phase 3 trial	Multi-countries	Multi-center	60	30	RCC 100%	X	V	X	CTCAE	Not report ICIs-AKI	488	4
Qin, 2022	2017 to 2020	Retrospective	China	Single-center	62	30.5	Lung 41.9%	X	V	X	KDIGO	1	551	65
Raghav, 2022	2016 to 2020	Prospective clinical Trial	U.S	Single-center	59	72	unknow primary advanced cancer	X	V	X	CTCAE	Not report ICIs-AKI	25	1

Study	Years of Data Collection	Study design	Country	Location	Mean Age	%, Female	Primary cancer type (%)	ICIs type			All-cause AKI definition	ICIs-related AKI definition	Number Enrolled	Number of all-cause AKI
								CTLA-4 inhibitor	PD-1 inhibitor	PD-L1 inhibitor				
Rao, 2022	2018 to 2020	Prospective clinical Trial	France	Multi-center	NR	64.9	Anal canal 100%	X	V	X	CTCAE	Not report ICIs-AKI	94	1
Reiss, 2022	2018 to 2021	Phase 1/2 trial	U.S	Single-center	64	41.7	Pancreatic cancer 100%	V	V	X	CTCAE	Not report ICIs-AKI	91	11
Rizvi, 2016	NA	Prospective clinical Trial	U.S	Multi-center	64	54	Lung 100%	X	V	X	CTCAE	Not report ICIs-AKI	56	8
Rodrigues, 2023	2017 to 2020	Prospective clinical Trial	France	Multi-center	47.9	100	Cervical cancer 100%	X	V	X	CTCAE	Not report ICIs-AKI	16	1
Rose, 2021	2016 to 2020	Prospective clinical Trial	U.S	Multi-center	66	18	UCC 100%	X	V	X	CTCAE	Not report ICIs-AKI	39	7
Rosenberg, 2023	2018 to 2019	Phase 2 trial	Multi-countries	Multi-center	75.5	27.9	UCC 100%	X	X	V	CTCAE	Not report ICIs-AKI	154	3
Seethapathy, 2019	2011 to 2016	Retrospective	U.S.	Single-center	63	39	Melanoma: 43%	V	V	V	AKI was defined as a >1.5-fold increase in creatinine from baseline within 12 months of checkpoint inhibitor initiation Sustained AKI: Cr remained >1.5 times the baseline for at least 3 days	1	1016	169
Seethapathy, 2020	2017 to 2018	Retrospective	U.S.	Single-center	65	50	Lung 43%	X	X	V	KDIGO AKI criteria & Sustained AKI was defined as AKI that lasted >48 hours	1	599	104
Seydel, 2021	2015 to 2019	Retrospective	Germany	Single-center	64.2	21.4	RCC 67.5%	V	V	V	CTCAE	Not report ICIs-AKI	126	5
Sezer, 2021	2017 to 2020	Prospective clinical Trial	Multi-countries	Multi-center	63	12	Lung 100%	X	V	X	CTCAE	Not report ICIs-AKI	355	2
Shah, 2020	2017 to 2018	Phase 2 trial	U.S	Multi-center	51	NA	Breast cancer 100%	X	V	X	CTCAE	Not report ICIs-AKI	30	1
Sharma, 2016 (CheckMate 032 trial)	2014 to 2015	Prospective clinical Trial	Multi-countries	Multi-center	65.5	31	UCC 100%	X	V	X	CTCAE	Not report ICIs-AKI	78	1
Shimamura, 2021	2015 to 2019	Retrospective	Japan	Single-center	67	25	Lung 63%	V	V	V	KDIGO	1	152	27
Sonpavde, 2023	NA	Prospective clinical Trial	U.S.	Multi-center	72.2	18.6	UCC 100%	X	X	V	CTCAE	Not report ICIs-AKI	43	4
Sorah, 2021	2014 to 2018	Retrospective	U.S.	Single-center	NR	NR	NR	V	V	V	Doubling or more of their baseline creatinine (KDIGO grade II AKI or greater)	1	1766	135

Study	Years of Data Collection	Study design	Country	Location	Mean Age	%, Female	Primary cancer type (%)	ICIs type			All-cause AKI definition	ICIs-related AKI definition	Number Enrolled	Number of all-cause AKI
								CTLA-4 inhibitor	PD-1 inhibitor	PD-L1 inhibitor				
Stein, 2020	2014 to 2018	Retrospective	France	Single-center	66.2	45	Melanoma: 100%	X	V	X	KDIGO	3	239	41
Sternberg, 2019	2016 to 2018	Phase 3 trial	Multi-countries	Multi-center	68	23	UCC 95%	X	X	V	CTCAE	Not report ICIs-AKI	997	18
Seylanova, 2021	2011 to 2020	Retrospective	U.K.	Single-center	NR	NR	NR	V	V	V	NR	3	1170	190
Stratigos, 2021	2017 to 2019	Phase 2 trial	Multi-countries	Multi-center	70	33	Basal cell carcinoma	X	V	X	CTCAE	Not report ICIs-AKI	84	2
Tanaka, 2020	2015 to 2020	Retrospective	Japan	Multi-center	69	21	RCC 100%	V	V	X	CTCAE	Not report ICIs-AKI	52	2
Tanizaki, 2022	2018 to 2019	Prospective clinical Trial	Japan	Single-center	65.6	60.7	unknow primary advanced cancer	X	V	X	CTCAE	Not report ICIs-AKI	56	2
Taylor, 2022	2015 to 2018	Phase 2 trial	U.S	Single-center	60.5	9.2	Head-Neck SqCC	X	V	X	CTCAE	Not report ICIs-AKI	76	3
Trevisani, 2021	2017 to 2018	Prospective clinical Trial	Italy	Multi-center	68	36	UCC 100%	X	V	X	KDIGO	Not report ICIs-AKI	143	1
Trevisani, 2022	2017 to 2020	Retrospective	Italy	Multi-center	72	36.4	Lung 100%	X	V	V	KDIGO	Not report ICIs-AKI	118	10
Tsung, 2023	2018 to 2021	Phase 2 trial	U.S	Single-center	71.5	11	UCC 100%	X	V	X	CTCAE	Not report ICIs-AKI	36	1
Tuscano, 2019	2012 to 2016	Phase 1 trial	U.S	Multi-center	62	27	Lymphoma 100%	V	X	X	CTCAE	Not report ICIs-AKI	33	1
Vano, 2022	2017 to 2019	Phase 2 trial	France	Multi-center	63.7	23.3	RCC 100%	V	V	X	CTCAE	Not report ICIs-AKI	159	1
Winer, 2021 (KEYNOTE-119)	2010 to 2020	Prospective clinical Trial	Multi-countries	Multi-center	50	100	Breast cancer 100%	X	V	X	CTCAE	Not report ICIs-AKI	309	0
Wise-Draper, 2022	2016 to 2020	Prospective clinical Trial	U.S.	Multi-center	59	30	Head-Neck SqCC	X	V	X	CTCAE	Not report ICIs-AKI	92	1
Wolchok, 2013	2009 to 2013	Prospective clinical Trial	U.S.	Single-center	60.3	41.8	Melanoma 100%	V	V	X	CTCAE	Not report ICIs-AKI	86	2
Yam, 2023	2016 to 2021	Phase 2 trial	U.S	Single-center	53.8	100	Breast cancer 100%	X	V	X	CTCAE	Not report ICIs-AKI	37	2
Yu, 2021	2017 to 2020	Prospective clinical Trial	Multi-countries	Multi-center	75	26	UCC 100%	X	V	V	CTCAE	Not report ICIs-AKI	89	5
Yu, 2022	2014 to 2019	Retrospective (match cohort for model prediction)	China	Single-center	NR	NR	NR	V	V	V	KDIGO	3	1616	111

Zinzani, 2019	NR	Prospective clinical Trial	Multi-countries	Multi-center	35.5	57	Lymphoma 100%	X	V	X	CTCAE	Not report ICIs-AKI	29	2

Abbreviation: AKI, acute kidney injury; AKIN, Acute Kidney Injury Network; CTCAE, Common Terminology Criteria for Adverse Events; CTLA-4: cytotoxic T lymphocyte-associated protein 4; eGFR, estimated Glomerular filtration rate; IQR, interquartile range; KDIGO, Kidney Disease: Improving Global Outcomes; NR, not reported; PD-1, programmed cell death protein 1; PD-L1, programmed cell death ligand 1; SCr, serum creatinine; RCC, Renal cell carcinoma; SD, standard deviation; UCC, Urothelial carcinoma; U.K., United Kingdom; U.S., United States

Footnote 1: The code for ICIs-related AKI definition: 1 (By nephrologists or physicians with or without biopsy), 2 (By prediction model with probability > 90%), 3 (By pre-defined serum creatinine criteria, and/or by nephrologists or physicians for exclusion of other causes of acute kidney injury, and/or responsiveness to steroid, and/or kidney replacement therapy, and/or renal biopsy), 4 (Studies did not report the definition of ICIs-related AKI). More detailed ICIs-related AKI definition was provided in **Table S4**.

Supplemental Table 4. Immune checkpoint inhibitors related AKI definition of the included studies

Study	ICIs related AKI definition
Abdelrahim, 2021<br>Espi, 2021<br>García-Carro, 2021<br>Gérard, 2022<br>Gu¨ ven, 2023<br>Kanbay, 2023<br>Knox, 2023<br>Liu, 2022<br>Meraz-Muñoz, 2020<br>Qin, 2022<br>Seethapathy, 2019<br>Seethapathy, 2020<br>Shimamura, 2021<br>Sorah, 2021	By nephrologists or physicians with or without biopsy
Baker, 2022	By prediction model with probability > 90%
Cortazar, 2020	By nephrologists or physicians + any of one : (1) doubling of SCr or (2) KRT
Gupta, 2021	By nephrologists or physicians + either of the following criteria:<br>(1) an increase in SCr ≥100% from baseline or KRT<br>(2) an increase in SCr ≥50% from baseline and at least one of the following: ATIN on kidney biopsy; ICIs therapy held for at least once cycle due to concern for ICIs -AKI; or treatment with corticosteroids due to concern for ICIs -AKI
Isik, 2021	ICIs-related AKI match any one:<br>(1) biopsy-confirmed; (2) kidney function was responsive to steroids; (3) progressed without steroids
Lumlertgul, 2023<br>Koks, 2021<br>Yu, 2022	Definite ICIs-AKI: biopsy confirm<br>Probable ICIs-AKI: meet three criteria (1) SCr > 1.5 baseline for two consecutive values or KRT; (2) absence of an alternative plausible cause; (3) at least one of three: sterile pyuria, eosinophilia, or recent or concomitant non-kidney irAE;<br>Possible ICIs-AKI: an alternative etiology was not readily attributable

Patel, 2021	By renal biopsy, when available, or clinically if a biopsy was not available.<br>Clinical inclusion criteria<br>(1) Cr rise after administration of ICI; (2) exclusion of other causes of acute kidney injury ; (3) improvement following systemic steroid administration
Seylanova, 2021	By nephrologists or physicians or by biopsy and any of one:<br>(1) an increase in SCr > 50% plus either tubulointerstitial nephritis on kidney biopsy, withholding ICIS, or steroids given due to AKI;<br>(2) a doubling of SCr from baseline or KRT
Stein, 2020	Possible ICIs-related AKI: the presence of an otherwise unexplained AKI, Not require a kidney biopsy, a favorable outcome after corticosteroids or the proof of a favorable evolution after drug discontinuation<br>Probable ICIs-related AKI: the presence of a steroid-sensitive AKI, improved after drug discontinuation, with urine analysis when available showing aseptic leucocyturia and/ or tubular proteinuria, with no other explanation for AKI, sometimes (but not necessarily) confirmed by kidney biopsy
Garcoa, 2023<br>Hoffman-Censitsm, 2020<br>Irwin, 2020<br>Ji, 2022<br>Lou, 2023	Not reported ICIs-related AKI definition

Abbreviation: AKI, acute kidney injury; ATIN, acute tubulointerstitial nephritis; ICIs, immune checkpoint inhibitors; irAE, immune-related adverse events; KRT, kidney replacement therapy; NR, not reported; SCr, serum creatinine.

Supplemental Table 5. Sensitivity analysis of PPI or drug exposure and the risk for all-cause or ICIs-related AKI development

**Exposure**	**All-cause AKI, OR (95% CI)**	**ICIs-related AKI, OR (95% CI)**
Random effect pairwise met-analysis (Knapp-Hartung method)		
PPI exposure	1.77 (1.43 to 2.18)	2.42 (1.96 to 2.97)
NSAID exposure	1.77 (1.10 to 2.83)	2.57 (1.68 to 3.93)
Multivariate meta-regression		
PPI exposure	1.74 (1.27 to 2.39)	2.78 (1.71 to 3.03)
NSAID exposure	1.79 (1.27 to 2.55)	2.58 (1.84 to 3.63)
Trim-and-fill method meta-analysis		
PPI exposure	1.48 (1.18 to 1.86)	2.42 (1.96 to 2.97)
NSAID exposure	2.49 (1.43 to 4.33)	2.57 (1.68 to 3.93)
Limited meta-analysis		
PPI exposure	1.71 (1.14 to 2.58)	2.91 (2.11 to 4.03)
NSAID exposure	2.22 (1.74 to 4.19)	2.99 (1.44 to 6.24)

Supplemental Table 6. Risk assessment of the included studies for pooled all-cause AKI occurrence rate

Study (Author, year)	Item 1[a]	Item 2[a, c]	Item 3 [a]	Item 4	Item 5	Item 6 [b]	Item 7 [b]	Item 8	Item 9	Item 10	Total Score	Risk
Abdelrahim, 2021	*		*	*	*	*	*	*	*	*	**9**	**Low**
Albiges, 2023				*	*	*	*	*	*	*	**7**	**Moderate**
Altman, 2016	*	*	*	*	*			*	*	*	**8**	**Low**
Antonia, 2019				*	*	*	*	*	*	*	**7**	**Moderate**
Antonia, 2019 (CheckMate 003, 017, 057, 063)				*	*	*	*	*	*	*	**7**	**Moderate**
Armand, 2020				*	*	*	*	*	*	*	**7**	**Moderate**
Ascierto, 2017				*	*	*	*	*	*	*	**7**	**Moderate**
Baggi, 2021 (milti or > 200)	*	*	*	*	*	*	*	*	*	*	**10**	**Low**
Baker, 2022	*	*	*	*	*	*	*	*	*	*	**10**	**Low**
Balanchivadze, 2021 (retro, single, <200)	*		*	*	*	*	*	*	*	*	**9**	**Low**
Balar, 2017 (KEYNOTE-052)				*	*	*	*	*	*	*	**7**	**Moderate**
Barata, 2020	*	*	*	*	*	*	*	*	*	*	**10**	**Low**
Bao, 2022	*	*	*	*	*	*	*	*	*	*	**10**	**Low**
Barlesi, 2018				*	*	*	*	*	*	*	**7**	**Moderate**
Basak, 2022				*	*	*	*	*	*	*	**7**	**Moderate**
Bellmunt, 2021 (IMvigor010 trial)				*	*	*	*	*	*	*	**7**	**Moderate**
Blanchette, 2022	*	*	*	*	*	*	*	*	*	*	**10**	**Low**

Brastianos, 2021				*	*	*	*	*	*	*	**7**	**Moderate**
Calabrò, 2018				*	*	*	*	*	*	*	**7**	**Moderate**
Carlo, 2022				*	*	*	*	*	*	*	**7**	**Moderate**
Cesne, 2019				*	*	*	*	*	*	*	**7**	**Moderate**
Chau, 2020				*	*	*	*	*	*	*	**7**	**Moderate**
Dang, 2022	*		*	*	*	*	*	*	*	*	**9**	**Low**
Desai, 2023				*	*	*	*	*	*	*	**7**	**Moderate**
Dizman, 2022				*	*	*	*	*	*	*	**7**	**Moderate**
Dirix, 2018				*	*	*	*	*	*	*	**7**	**Moderate**
D'Souza, 2019				*	*	*	*	*	*	*	**7**	**Moderate**
Dummer, 2023				*	*	*	*	*	*	*	**7**	**Moderate**
Egelston, 2023				*	*	*	*	*	*	*	**7**	**Moderate**
Espi, 2021	*	*	*	*	*	*	*	*	*	*	**10**	**Low**
Fedorova, 2022	*		*	*	*	*	*	*	*	*	**9**	**Low**
Feun, 2019				*	*	*	*	*	*	*	**7**	**Moderate**
García-Carro, 2022	*	*	*	*	*	*	*	*	*	*	**10**	**Low**
Gettinger, 2021				*	*	*	*	*	*	*	**7**	**Moderate**
Giglio, 2022	*		*	*	*	*	*	*	*	*	**9**	**Low**
Garcoa, 2023 (retro, > 200)	*	*	*	*	*	*	*	*	*	*	**10**	**Low**
Garon, 2020				*	*	*	*	*	*	*	**7**	**Moderate**
Goldberg, 2016				*	*			*	*	*	**5**	**High**
Goldberg, 2020				*	*	*	*	*	*	*	**7**	**Moderate**

Grimm, 2022 (pros, obeser)	*	*	*	*	*	*	*	*	*	*	**10**	**Low**
Gulati, 2023				*	*	*	*	*	*	*	**7**	**Moderate**
Guven, 2023	*	*	*	*	*	*	*	*	*	*	**10**	**Low**
Hellmann, 2017 (CheckMate 012)				*	*	*	*	*	*	*	**7**	**Moderate**
Heppt, 2016 (mul, no aki)	*	*	*	*	*			*	*	*	**8**	**Low**
Herbst, 2019				*	*	*	*	*	*	*	**7**	**Moderate**
Hoffman-Censitsm, 2020				*	*	*	*	*	*	*	**7**	**Moderate**
Isik, 2021	*	*	*	*	*	*	*	*	*	*	**10**	**Low**
Irwin, 2020	*	*	*	*	*			*	*	*	**8**	**Low**
Ji, 2022	*	*	*	*	*	*	*	*	*	*	**10**	**Low**
Kanbay, 2023	*	*	*	*	*	*	*	*	*	*	**10**	**Low**
Kaufman, 2016				*	*			*	*	*	**5**	**High**
Koks, 2021	*	*	*	*	*	*	*	*	*	*	**10**	**Low**
Knox, 2023	*	*	*	*	*	*	*	*	*	*	**10**	**Low**
Kuruvilla, 2021				*	*	*	*	*	*	*	**7**	**Moderate**
Langer, 2016				*	*	*	*	*	*	*	**7**	**Moderate**
Lee, 2020				*	*	*	*	*	*	*	**7**	**Moderate**
Leon, 2020	*	*	*	*	*	*	*	*	*	*	**10**	**Low**
Li, 2023				*	*	*	*	*	*	*	**7**	**Moderate**
Liu, 2022	*	*	*	*	*	*	*	*	*	*	**10**	**Low**
Long, 2019 (KEYNOTE-252)				*	*	*	*	*	*	*	**7**	**Moderate**
Lou, 2023	*	*	*	*	*	*	*	*	*	*	**10**	**Low**

Lumlertgul, 2023	*	*	*	*	*	*	*	*	*	*	**10**	**Low**
Manohar, 2018	*		*	*	*	*	*	*	*	*	**9**	**Low**
Malhotra, 2021				*	*	*	*	*	*	*	**7**	**Moderate**
Martin, 2020	*	*	*	*	*	*	*	*	*	*	**10**	**Low**
Massard, 2016				*	*	*	*	*	*	*	**7**	**Moderate**
Mateos, 2019				*	*	*	*	*	*	*	**7**	**Moderate**
Matthew 2019				*	*	*	*	*	*	*	**7**	**Moderate**
Meraz-Muñoz, 2020	*	*	*	*	*	*	*	*	*	*	**10**	**Low**
Middleton, 2020				*	*			*	*	*	**5**	**High**
Moreau, 2021				*	*	*	*	*	*	*	**7**	**Moderate**
Motzer, 2019				*	*	*	*	*	*	*	**7**	**Moderate**
Mushtaq, 2019	*	*	*	*	*	*	*	*	*	*	**10**	**Low**
Oh, 2022				*	*	*	*	*	*	*	**7**	**Moderate**
Oleas, 2021	*	*	*	*	*	*	*	*	*	*	**10**	**Low**
Overman, 2017				*	*	*	*	*	*	*	**7**	**Moderate**
Patel, 2021	*		*	*	*	*	*	*	*	*	**9**	**Low**
Patel, 2020				*	*	*	*	*	*	*	**7**	**Moderate**
Paz-Ares, 2021				*	*	*	*	*	*	*	**7**	**Moderate**
Powles, 2020				*	*	*	*	*	*	*	**7**	**Moderate**
Powles, 2022				*	*	*	*	*	*	*	**7**	**Moderate**
Qin, 2022	*	*	*	*	*	*	*	*	*	*	**10**	**Low**
Raghav, 2022				*	*	*	*	*	*	*	**7**	**Moderate**

Rao, 2022				*	*	*	*	*	*	*	**7**	**Moderate**
Reiss, 2022				*	*	*	*	*	*	*	**7**	**Moderate**
Rizvi, 2016				*	*	*	*	*	*	*	**7**	**Moderate**
Rodrigues, 2023				*	*	*	*	*	*	*	**7**	**Moderate**
Rose, 2021				*	*	*	*	*	*	*	**7**	**Moderate**
Rosenberg, 2023				*	*	*	*	*	*	*	**7**	**Moderate**
Seethapathy, 2019	*	*	*	*	*	*	*	*	*	*	**10**	**Low**
Seethapathy, 2020	*	*	*	*	*	*	*	*	*	*	**10**	**Low**
Seydel, 2021	*		*	*	*	*	*	*	*	*	**9**	**Low**
Sezer, 2021				*	*	*	*	*	*	*	**7**	**Moderate**
Shah, 2020				*	*	*	*	*	*	*	**7**	**Moderate**
Sharma, 2016 (CheckMate 032 trial)				*	*	*	*	*	*	*	**7**	**Moderate**
Sonpavde, 2023				*	*	*	*	*	*	*	**7**	**Moderate**
Sorah, 2021	*	*	*	*	*	*	*	*	*	*	**10**	**Low**
Stein, 2020	*	*	*	*	*	*	*	*	*	*	**10**	**Low**
Sternberg, 2019				*	*	*	*	*	*	*	**7**	**Moderate**
Seylanova, 2021	*	*	*	*	*			*	*	*	**8**	**Low**
Stratigos, 2021				*	*	*	*	*	*	*	**7**	**Moderate**
Tanaka, 2020	*	*	*	*	*	*	*	*	*	*	**10**	**Low**
Tanizaki, 2022				*	*	*	*	*	*	*	**7**	**Moderate**
Taylor, 2022				*	*	*	*	*	*	*	**7**	**Moderate**

Trevisani, 2021				*	*	*	*	*	*	*	**7**	**Moderate**
Trevisani, 2022	*	*	*	*	*	*	*	*	*	*	**10**	**Low**
Tsung, 2023				*	*	*	*	*	*	*	**7**	**Moderate**
Tuscano, 2019				*	*	*	*	*	*	*	**7**	**Moderate**
Vano, 2022				*	*	*	*	*	*	*	**7**	**Moderate**
Winer, 2021 (KEYNOTE-119)				*	*	*	*	*	*	*	**7**	**Moderate**
Wise-Draper, 2022				*	*	*	*	*	*	*	**7**	**Moderate**
Wolchok, 2013				*	*	*	*	*	*	*	**7**	**Moderate**
Yam, 2023				*	*	*	*	*	*	*	**7**	**Moderate**
Yu, 2021				*	*	*	*	*	*	*	**7**	**Moderate**
Yu, 2022	*	*	*	*	*	*	*	*	*	*	**10**	**Low**
Zinzani, 2019				*	*	*	*	*	*	*	**7**	**Moderate**

[a] For prospective clinical studies, item related to close representation to the target population is in concern

[b] For study without clear definition of acute kidney injury, this item is consider as high risk

[c] For retrospective with sample size less than 200 and from single center study, concern about true representation to target population is in concern

Supplemental Table 7. Risk assessment of the included studies for pooled ICIs-related AKI occurrence rate

Study (Author, year)	Item 1[a]	Item 2[a, b]	Item 3[a]	Item 4	Item 5	Item 6[c]	Item 7[d]	Item 8	Item 9	Item 10	Total Score	Risk
Abdelrahim, 2021	*	*	*	*	*	*		*	*	*	**9**	**Low**
Espi, 2021	*	*	*	*	*	*		*	*	*	**9**	**Low**
García-Carro, 2022	*	*	*	*	*	*		*	*	*	**9**	**Low**
Garcoa, 2023	*	*	*	*	*			*	*	*	**8**	**Low**
Guven, 2023	*	*	*	*	*	*		*	*	*	**9**	**Low**
Hoffman-Censitsm, 2020				*	*			*	*	*	**5**	**High**
Irwin, 2020	*	*	*	*	*			*	*	*	**8**	**Low**
Isik, 2021	*	*	*	*	*	*	*	*	*	*	**10**	**Low**
Liu, 2022	*	*	*	*	*	*		*	*	*	**9**	**Low**
Lou, 2023	*	*	*	*	*			*	*	*	**8**	**Low**
Lumlertgul, 2023	*	*	*	*	*	*	*	*	*	*	**10**	**Low**
Ji, 2022	*	*	*	*	*			*	*	*	**8**	**Low**
Koks, 2021	*	*	*	*	*	*	*	*	*	*	**10**	**Low**
Kanbay, 2023	*	*	*	*	*	*		*	*	*	**9**	**Low**
Knox, 2023	*	*	*	*	*	*	*	*	*	*	**10**	**Low**
Meraz-Muñoz, 2020	*	*	*	*	*	*		*	*	*	**9**	**Low**
Patel, 2021	*		*	*	*	*	*	*	*	*	**9**	**Low**
Qin, 2022	*	*	*	*	*	*		*	*	*	**9**	**Low**
Seethapathy, 2019	*	*	*	*	*	*		*	*	*	**9**	**Low**

Seethapathy, 2020	*	*	*	*	*	*		*	*	*	**9**	**Low**
Shimamura, 2021	*		*	*	*	*		*	*	*	**8**	**Low**
Sorah, 2021	*	*	*	*	*	*		*	*	*	**9**	**Low**
Stein, 2020	*	*	*	*	*	*	*	*	*	*	**10**	**Low**
Seylanova, 2021	*	*	*	*	*	*	*	*	*	*	**10**	**Low**
Yu, 2022	*	*	*	*	*	*	*	*	*	*	**10**	**Low**

[a] For prospective clinical studies, item related to close representation to the target population is in concern

[b] For retrospective with sample size less than 200 and from single center study, concern about true representation to target population is in concern

[c] For study without reported definition of ICIs-related acute kidney injury, this item is consider as high risk

[d] For study without pre-defined, specific definition of ICIs-related acute kidney injury, this item is consider as high risk

Supplemental Table 8. Risk assessment by Newcastle-Ottawa scale regarding drug exposure and all-cause AKI development

PPIs/NSAID exposure and the AKI development: cohort studies										
	SELECTION				COMPARABILITY	OUTCOME			NOS score	Risk of bias
Study	Representativeness of the exposed cohort (1)	Selection of the non-exposed cohort (1)	Ascertainment of exposure (1)	Demonstration that outcome of interest was not present at start of study (1)	Comparability of cohorts on the basis of the design or analysis (2)	Assessment of outcome (1)	Was follow-up long enough for outcomes to occur (1)	Adequacy of follow up of cohorts (1)		
Abdelrahim, 2021	1	1	0[a]	1	0	1	1	1	**6**	**H**
Giglio, 2022	1	1	1	1	0	1	1	1	**7**	**L**
Guven, 2023	1	1	1	1	0	1	1	1	**7**	**L**
Ji, 2022	1	1	1	1	0	1	1	1	**7**	**L**
Kanbay, 2023	1	1	1	1	0	1	1	1	**7**	**L**
Koks, 2021	1	1	1	1	0	1	1	1	**7**	**L**
Liu, 2022	1	1	1	1	0	1	1	1	**7**	**L**
Lumlertgul, 2023	1	1	1	1	0	1	1	1	**7**	**L**
Meraz-Muñoz, 2020	1	1	1	1	0	1	1	1	**7**	**L**
Qin, 2022	1	1	1	1	0	1	1	1	**7**	**L**
Seethapathy, 2019	1	1	1	1	0	1	1	1	**7**	**L**
Seethapathy, 2020	1	1	1	1	0	1	1	1	**7**	**L**

Stein, 2020	1	1	1	1	0	1	1	1	**7**	**L**

Footnote: A study can be awarded a maximum of one point for each item within the Selection and Outcome categories if it is high quality. A maximum of two points can be given for Comparability. The Newcastle-Ottawa scale have total 8 items within 3 domain and the total maximum score is 9. A study has a score from 7-9 was consider as high quality, a score from 4-6 was consider as high risk, and a score from 0-3 was consider as very high risk of bias.

[a]Not all participants in the analysis have information about PPI or NSAID exposure or not

Supplemental Table 9. Risk assessment by Newcastle-Ottawa scale regarding drug exposure and ICIs-related AKI development

PPIs/NSAID exposure and the AKI development: cohort studies										
	SELECTION				COMPARABILITY	OUTCOME			NOS score	Risk of bias
**Study**	**Representativeness of the exposed cohort (1)**	**Selection of the non-exposed cohort (1)**	**Ascertainment of exposure (1)**	**Demonstration that outcome of interest was not present at start of study (1)**	**Comparability of cohorts on the basis of the design or analysis (2)**	**Assessment of outcome (1)**	**Was follow-up long enough for outcomes to occur (1)**	**Adequacy of follow up of cohorts (1)**		
Espi, 2021	1	1	1	1	0	1	1	1	**7**	**L**
Liu, 2022	1	1	1	1	0	1	1	1	**7**	**L**
Lumlertgul, 2023	1	1	1	1	0	1	1	1	**7**	**L**
Lou, 2023	1	1	1	1	0	0	1	1	**6**	**H**
Qin, 2022	1	1	1	1	0	1	1	1	**7**	**L**
Seethapathy, 2019	1	1	1	1	0	1	1	1	**7**	**L**
Seethapathy, 2020	1	1	1	1	0	1	1	1	**7**	**L**
Shimamura, 2021	1	1	1	1	0	1	1	1	**7**	**L**
**PPIs/NSAID exposure and the AKI development: case-control studies**										
	**SELECTION**				**COMPARABILITY**	**OUTCOME**			**NOS score**	**Risk of bias**
**Study**	**Is the case definition adequate (1)**	**Representativeness of the cases (1)**	**Selection of Controls (1)**	**Definition of Controls (1)**	**Comparability of cases and controls on the basis of the design or analysis (2)**	**Assessment of outcome (1)**	**Same method of ascertainment for cases and controls (1)**	**Non-Response rates (1)**		

Cortazar, 2020	1	1	1	1	0	1	1	0	**6**	**H**
Gérard, 2022	1	1	1	1	0	0[a]	1	0	**5**	**H**
Gupta, 2021	1	1	1	1	0	1	1	0	**6**	**H**
Yu, 2022	1	1	1	1	0	1	1	0	**6**	**H**

Footnote: A study can be awarded a maximum of one point for each item within the Selection and Outcome categories if it is high quality. A maximum of two points can be given for Comparability. The Newcastle-Ottawa scale have total 8 items within 3 domain and the total maximum score is 9. A study has a score from 7-9 was consider as high quality, a score from 4-6 was consider as high risk, and a score from 0-3 was consider as very high risk of bias.

[a] Without AKI definition and no pre-specific ICIs-related AKI definition

Supplemental Table 10. Risk assessment of the included cohort studies for AKI and mortality

**Mortality in ICIs recipients with AKI**						
**Study**	**Study participation**	**Study Attrition**	**Prognostic Factor Measurement (ICIs exposure)**	**Outcome Measurement**	**Study Confounding**	**Statistical Analysis and Reporting**
Baker, 2022	Low	Low	Low	Low	Low	Low
Bao, 2022	Moderate	Low	Low	Low	Low	Low
García-Carro, 2022	Moderate	Low	Low	Low	Low	Low
Ji, 2022	Moderate	Low	Low	Low	Low	Low
Seethapathy, 2020	Moderate	Low	Low	Low	Low	Low
Shimamura, 2021	Moderate	Low	Low	Moderate	Low	Low
Liu, 2022	Moderate	Low	Low	Low	Low	Low
Lumlertgul, 2023	Low	Low	Low	Low	Low	Low

Footnote: Single-center was ranked as moderate risk of bias in study participation domain. For study without known follow up period regarding mortality outcome, moderate risk was judged in outcome measurement domain.

Supplemental Table 11. GRADE table summarizing the quality of the evidence for PPIs exposure and AKI development

Certainty assessment							№ of patients		Effect		Certainty	Importance
**№ of studies**	**Study design**	**Risk of bias**	**Inconsistency**	**Indirectness**	**Imprecision**	**Other considerations**	**PPI**	**non-PPI exposure**	**Relative (95% CI)**	**Absolute (95% CI)**		
**All-cause of AKI**												
13	observational studies	serious[a]	not serious	not serious	not serious	strong association	502/3998 (12.6%)	386/4557 (8.5%)	OR 1.77 (1.43 to 2.18)	56 more per 1,000 (from 32 more to 83 more)	⨁⨁◯◯ Low	IMPORTANT
**ICIs related AKI**												
12	observational studies	serious[a]	not serious	not serious	not serious	strong association	591/4207 (14.0%)	460/3578 (12.9%)	OR 2.42 (1.96 to 2.97)	135 more per 1,000 (from 96 more to 176 more)	⨁⨁◯◯ Low	IMPORTANT

Abbreviation: CI: confidence interval; OR: odds ratio

Explanations

a. Potential baseline imbalance noted from PPIs exposure and PPIs non-exposure group in cohort studies and possible outcome reporting bias

Supplemental Table 12. GRADE table summarizing the quality of the evidence for NSAID exposure and AKI development

Certainty assessment							№ of patients		Effect		Certainty	Importance
**№ of studies**	**Study design**	**Risk of bias**	**Inconsistency**	**Indirectness**	**Imprecision**	**Other considerations**	**NSAID**	**non-exposure**	**Relative (95% CI)**	**Absolute (95% CI)**		
**All-cause AKI**												
11	observational studies	serious[a]	serious[b]	not serious	not serious	none	248/1884 (13.2%)	570/6330 (9.0%)	OR 1.77 (1.10 to 2.83)	59 more per 1,000 (from 8 more to 129 more)	⨁◯◯◯ Very low	IMPORTANT
**ICIs-related AKI**												
10	observational studies	serious[a]	not serious	not serious	not serious	strong association	200/2017 (9.9%)	284/4496 (6.3%)	OR 2.57 (1.68 to 3.93)	85 more per 1,000 (from 39 more to 146 more)	⨁⨁◯◯ Low	IMPORTANT

Abbreviation: CI: confidence interval; OR: odds ratio

Explanations

a. Potential imbalance in baseline. The ICIs-related AKI definitions were different and some studies did not have pre-defined criteria. Possible outcome reporting bias should also be taken into consideration.

b. High heterogeneity

Supplemental Table 13. GRADE table summarizing the quality of the evidence regarding AKI as prognostic factor

**Outcome: Mortality; Prognostic factors: AKI**										
Number of participants	Number of studies	Number of cohorts	Estimated effect size (95% CI)	GRADE factors						
				Study limitations	Inconsistency	Indirectness	Imprecision	Publication bias	Other considerations (Moderate/large effect size; Dose effect)	Overall quality
6435	8	8	Odds ratio 1.71 (1.06 to 2.94)	serious	serious	not serious	not serious	Not serious	none	Very Low

Abbreviation: CI: confidence interval; OR: odds ratio

Supplemental Figure 1. PRISMA Flow Diagram

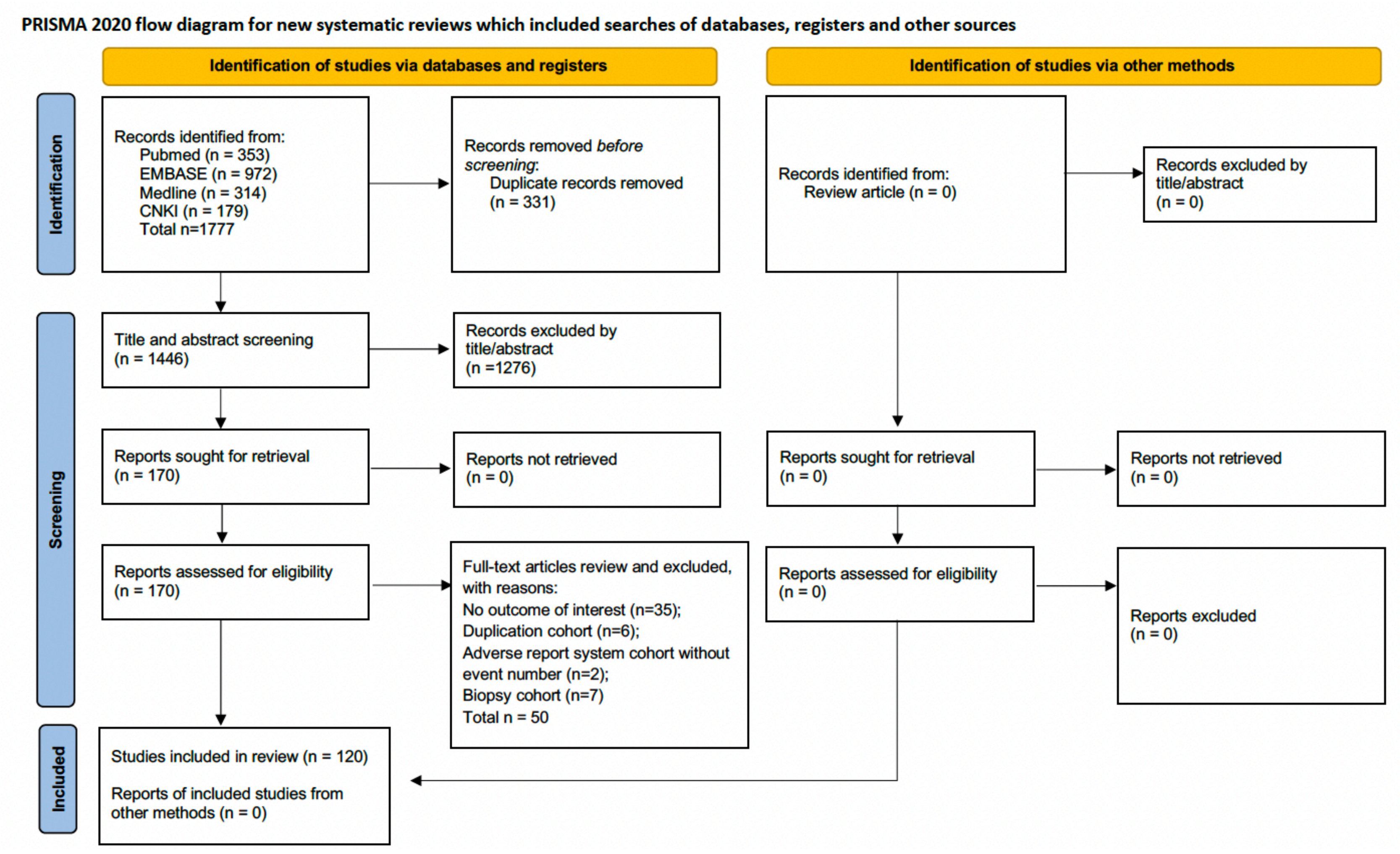

Supplemental Figure 2. Subgroup analysis of pooled occurrence rate of all-cause AKI

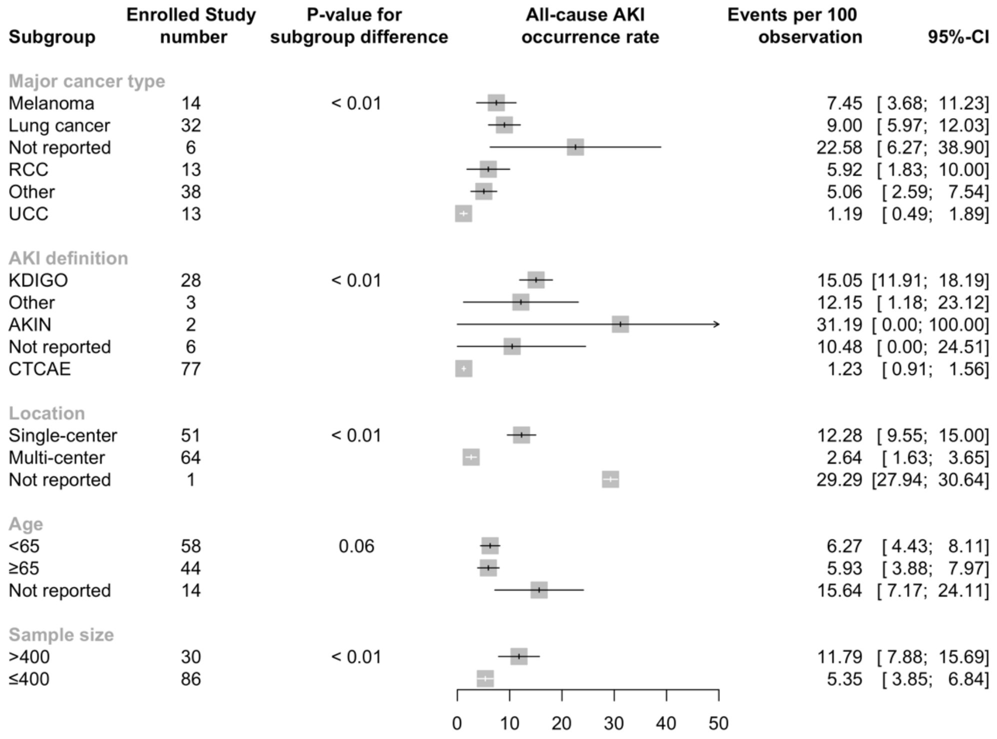

Supplemental Figure 3. Subgroup analysis of pooled occurrence rate of ICI-related AKI

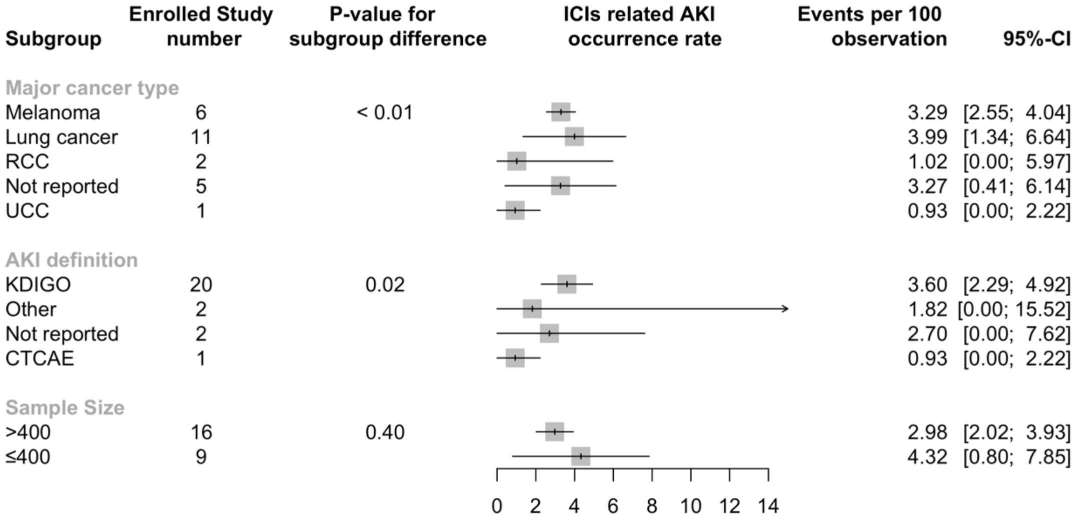

Supplemental Figure 4. Pooled occurrence rate of severe all-cause AKI

Study	Events	Total	Events per 100 observations	95%-CI	Weight
**Study Deisgn = Retrospective**					
Baggi, 2021	0	131	0.00	[0.00; 2.78]	1.7%
Fedorova, 2022	0	21	0.00	[0.00; 16.11]	0.4%
Martin, 2020	0	109	0.00	[0.00; 3.33]	1.7%
Ji, 2022	27	1615	1.67	[1.10; 2.42]	1.9%
Tanaka, 2020	1	52	1.92	[0.05; 10.26]	0.8%
Liu, 2022	7	305	2.30	[0.93; 4.67]	1.5%
Dang, 2022	2	78	2.56	[0.31; 8.96]	0.9%
Stein, 2020	7	239	2.93	[1.19; 5.94]	1.3%
Espi, 2021	11	352	3.12	[1.57; 5.52]	1.5%
Koks, 2021	23	676	3.40	[2.17; 5.06]	1.6%
Knox, 2023	16	449	3.56	[2.05; 5.72]	1.5%
Qin, 2022	20	551	3.63	[2.23; 5.55]	1.6%
Barata, 2020	1	27	3.70	[0.09; 18.97]	0.3%
Giglio, 2022	4	89	4.49	[1.24; 11.11]	0.7%
Guven, 2023	12	252	4.76	[2.48; 8.17]	1.2%
García-Carro, 2022	49	759	6.46	[4.81; 8.45]	1.5%
Blanchette, 2022	289	4380	6.60	[5.88; 7.37]	1.8%
Seethapathy, 2019	72	1016	7.09	[5.59; 8.84]	1.6%
Kanbay, 2023	17	235	7.23	[4.27; 11.33]	0.9%
Meraz-Muñoz, 2020	24	309	7.77	[5.04; 11.34]	1.0%
Manohar, 2018	11	134	8.21	[4.17; 14.21]	0.6%
Random effects model		11779	3.82	[2.68; 4.97]	26.2%
Heterogeneity: I^2 = 91% [88%; 94%]					
**Study Deisgn = Prospective clinical trial**					
Goldberg, 2020	0	42	0.00	[0.00; 8.41]	1.0%
Trevisani, 2021	0	143	0.00	[0.00; 2.55]	1.8%
Calabrò, 2018	0	40	0.00	[0.00; 8.81]	0.9%
Feun, 2019	0	28	0.00	[0.00; 12.34]	0.6%
Sezer, 2021	0	355	0.00	[0.00; 1.03]	1.9%
Winer, 2021	0	309	0.00	[0.00; 1.19]	1.9%
Egelston, 2023	0	10	0.00	[0.00; 30.85]	0.1%
Oh, 2022	0	33	0.00	[0.00; 10.58]	0.8%
Ascierto, 2017	1	726	0.14	[0.00; 0.77]	1.9%
Antonia, 2019 (CheckMate 003, 017, 057, 063)	1	664	0.15	[0.00; 0.84]	1.9%
Dummer, 2023	1	533	0.19	[0.00; 1.04]	1.9%
Barlesi, 2018	1	396	0.25	[0.01; 1.40]	1.9%
Balar, 2017	1	370	0.27	[0.01; 1.50]	1.9%
Long, 2019	2	705	0.28	[0.03; 1.02]	1.9%
Antonia, 2019	1	304	0.33	[0.01; 1.82]	1.9%
Gettinger, 2021	1	247	0.40	[0.01; 2.23]	1.8%
Powles, 2022	2	488	0.41	[0.05; 1.47]	1.9%
Powles, 2020	3	685	0.44	[0.09; 1.27]	1.9%
Hoffman-Censitsm, 2020	1	214	0.47	[0.01; 2.58]	1.8%
Bellmunt, 2021	2	390	0.51	[0.06; 1.84]	1.8%
Dirix, 2018	1	168	0.60	[0.02; 3.27]	1.7%
Vano, 2022	1	159	0.63	[0.02; 3.45]	1.7%
Albiges, 2023	1	158	0.63	[0.02; 3.48]	1.7%
Armand, 2020	1	137	0.73	[0.02; 4.00]	1.6%
Motzer, 2019	4	547	0.73	[0.20; 1.86]	1.8%
Paz-Ares, 2021	3	358	0.84	[0.17; 2.43]	1.8%
Rao, 2022	1	94	1.06	[0.03; 5.79]	1.4%
Wise-Draper, 2022	1	92	1.09	[0.03; 5.91]	1.4%
Herbst, 2019	1	92	1.09	[0.03; 5.91]	1.4%
Reiss, 2022	1	91	1.10	[0.03; 5.97]	1.3%
Sternberg, 2019	11	997	1.10	[0.55; 1.97]	1.9%
Stratigos, 2021	1	84	1.19	[0.03; 6.46]	1.3%
Sharma, 2016	1	78	1.28	[0.03; 6.94]	1.2%
Hellmann, 2017	1	77	1.30	[0.03; 7.02]	1.2%
Rosenberg, 2023	2	154	1.30	[0.16; 4.61]	1.5%
Kuruvilla, 2021	2	148	1.35	[0.16; 4.80]	1.5%
Overman, 2017	1	74	1.35	[0.03; 7.30]	1.2%
Matthew 2019	2	137	1.46	[0.18; 5.17]	1.4%
Mateos, 2019	1	62	1.61	[0.04; 8.66]	1.0%
Massard, 2016	1	61	1.64	[0.04; 8.80]	1.0%
Garon, 2020	8	405	1.98	[0.86; 3.85]	1.6%
Wolchok, 2013	2	86	2.33	[0.28; 8.15]	1.0%
Desai, 2023	1	39	2.56	[0.06; 13.48]	0.6%
Rose, 2021	1	39	2.56	[0.06; 13.48]	0.6%
Goldberg, 2016	1	36	2.78	[0.07; 14.53]	0.5%
Tsung, 2023	1	36	2.78	[0.07; 14.53]	0.5%
Gulati, 2023	1	35	2.86	[0.07; 14.92]	0.5%
Tuscano, 2019	1	33	3.03	[0.08; 15.76]	0.5%
Patel, 2020 (b)	1	32	3.12	[0.08; 16.22]	0.4%
Shah, 2020	1	30	3.33	[0.08; 17.22]	0.4%
Langer, 2016	2	59	3.39	[0.41; 11.71]	0.6%
Zinzani, 2019	1	29	3.45	[0.09; 17.76]	0.4%
D'Souza, 2019	1	29	3.45	[0.09; 17.76]	0.4%
Chau, 2020	1	28	3.57	[0.09; 18.35]	0.4%
Tanizaki, 2022	2	56	3.57	[0.44; 12.31]	0.6%
Lee, 2020	1	26	3.85	[0.10; 19.64]	0.3%
Raghav, 2022	1	25	4.00	[0.10; 20.35]	0.3%
Yu, 2021	4	89	4.49	[1.24; 11.11]	0.7%
Rizvi, 2016	3	56	5.36	[1.12; 14.87]	0.5%
Yam, 2023	2	37	5.41	[0.66; 18.19]	0.3%
Brastianos, 2021	1	18	5.56	[0.14; 27.29]	0.2%
Cesne, 2019	1	16	6.25	[0.16; 30.23]	0.1%
Rodrigues, 2023	1	16	6.25	[0.16; 30.23]	0.1%
Rodrigues, 2023	1	16	6.25	[0.16; 30.23]	0.1%
Dizman, 2022	2	29	6.90	[0.85; 22.77]	0.2%
Li, 2023	1	12	8.33	[0.21; 38.48]	0.1%
Moreau, 2021	1	10	10.00	[0.25; 44.50]	0.1%
Random effects model		11772	0.37	[0.25; 0.49]	72.1%
Heterogeneity: I^2 = 0% [0%; 29%]					
**Study Deisgn = Prospective observational**					
Grimm, 2022	2	228	0.88	[0.11; 3.13]	1.7%
**Random effects model**		**23779**	**1.79**	**[1.36; 2.21]**	**100.0%**
Heterogeneity: I^2 = 85% [82%; 87%]					
Test for subgroup differences: $p < 0.01$					

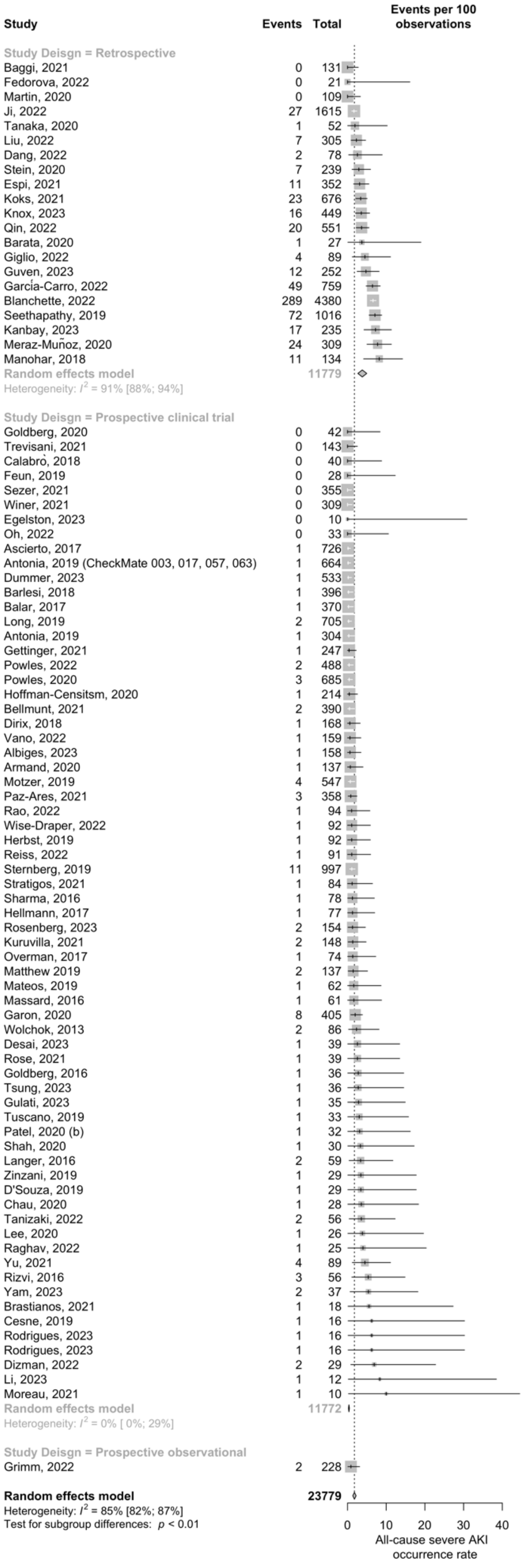

Supplemental Figure 5. Pooled occurrence rate of severe ICIs-related AKI

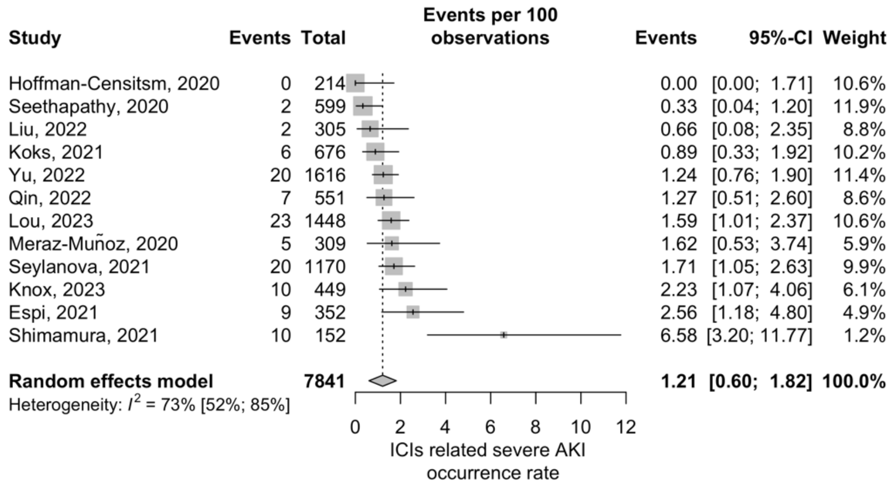

Supplemental Figure 6. Pooled occurrence rate of dialysis-requiring all-cause AKI

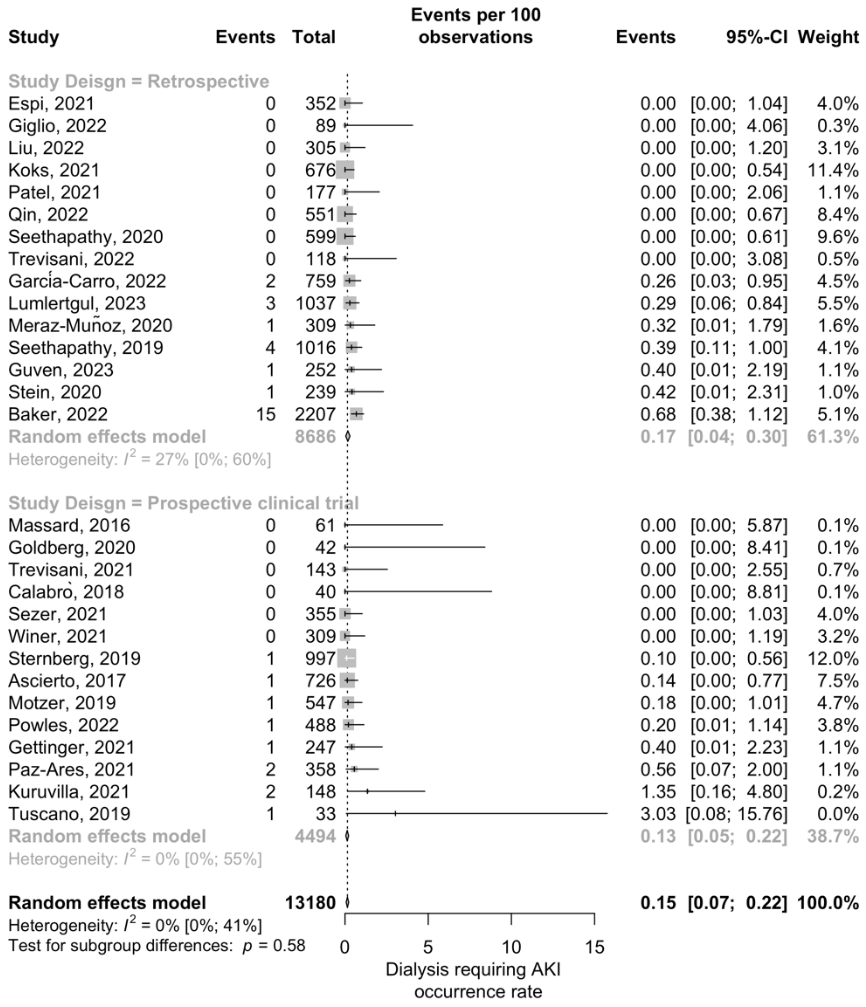

Supplemental Figure 7. Pooled occurrence rate of dialysis-requiring ICIs-related AKI

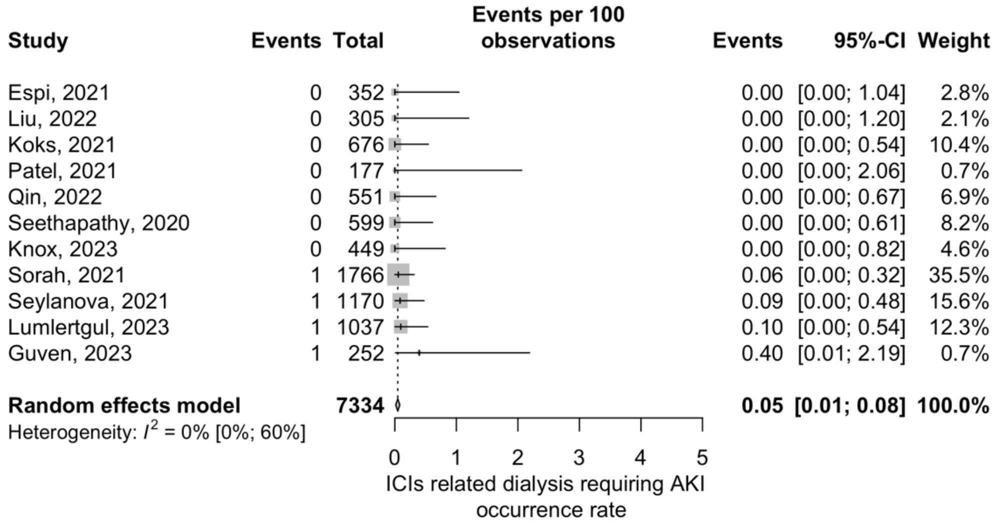

Supplemental Figure 8. Forest plot of AKI and the risk of mortality

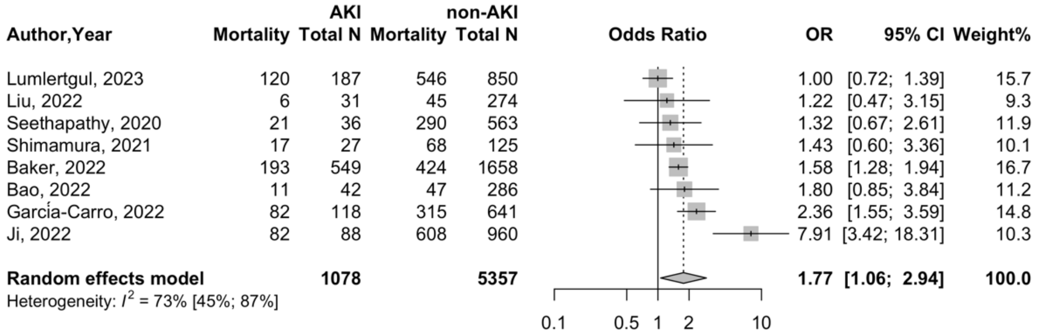

Supplemental Figure 9. Funnel plot for PPI or NSAID exposure and development of all-cause or ICIs-related AKI

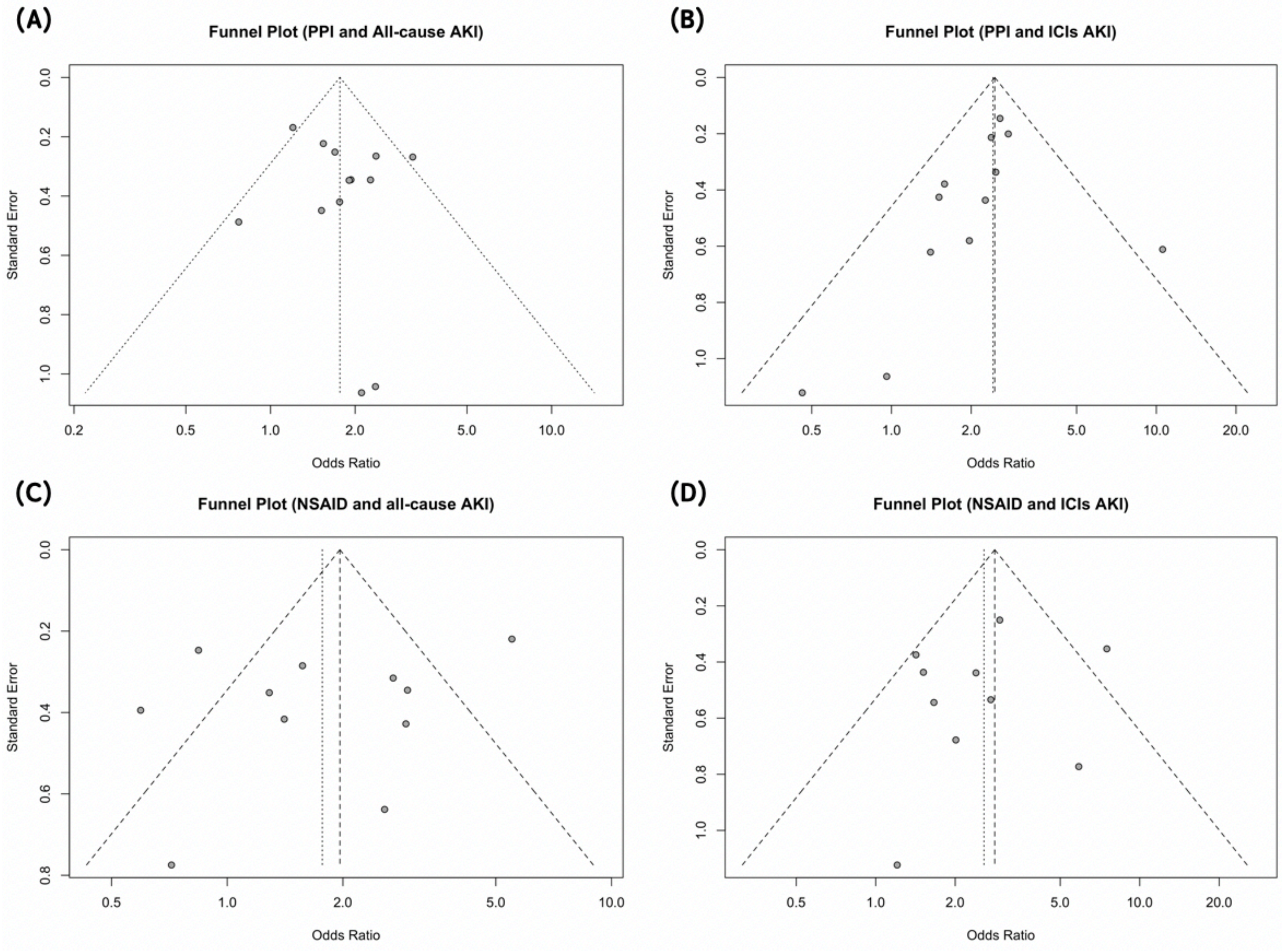

Supplemental Document 1

For examining the associations between the use of PPI or NSAID and the incidence of AKI, we also conducted multivariate meta-regression by using linear mixed effects model with the restricted maximum likelihood estimation. The statistical analyses were undertaken by using the "*rma.mv*" function in the metafor package for R software. We included the use of PPI or NSAID as fixed effects and the study-by-treatment interaction as random effects. Some case-control studies reported AKI events in patients with or without PPI and also in those with or without NSAID, separately. Because patients were counted twice in those studies, we halved the reported AKI case and total numbers of PPI or NSAID patients and their controls in our analyses and included study level as random effects to account for potential correlations within the two groups.

In the multivariate meta-regression analysis, both PPI and NSAID was associated with increased odds ratio for all-cause AKI development (PPI: OR=1.74, 95% CI= 1.27 to 2.39; NSAID: OR=1.79, 95% CI= 1.27 to 2.55) in comparison with non-exposure group. Both PPI and NSAID was associated with increased odds ratio for ICIs-related AKI development (PPI: OR=2.78, 95% CI= 1.71 to 3.03; NSAID: OR=2.58, 95% CI= 1.68 to 3.93) in comparison with non-exposure group. (**Table S5**)

Supplemental Document 2

Item	Criteria for answers (please circle one option)
**External validity**	
1. Was the study’s target population a close representation of the national population in relation to relevant variables, e.g. age, sex, occupation?	☐ Yes (LOW RISK): The study’s target population was a close representation of the national population.<br>☐ No (HIGH RISK): The study’s target population was clearly NOT representative of the national population.
2. . Was the sampling frame a true or close representation of the target population?	☐ Yes (LOW RISK): The sampling frame was a true or close representation of the target population.<br>☐ No (HIGH RISK): The sampling frame was NOT a true or close representation of the target population.
3. Was some form of random selection used to select the sample, OR, was a census undertaken?	☐ Yes (LOW RISK): A census was undertaken, OR, some form of random selection was used to select the sample (e.g. simple random sampling, stratified random sampling, cluster sampling, systematic sampling).<br>☐ No (HIGH RISK): A census was NOT undertaken, AND some form of random selection was NOT used to select the sample
4. Was the likelihood of non-response bias minimal?	☐ Yes (LOW RISK): The response rate for the study was >/=75%, OR, an analysis was performed that showed no significant difference in relevant demographic characteristics between responders and non-responders<br>☐ No (HIGH RISK): The response rate was <75%, and if any analysis comparing responders and non-responders was done, it showed a significant difference in relevant demographic characteristics between responders and non-responders.
**Internal validity**	
5. Were data collected directly from the subjects (as opposed to a proxy)?	☐Yes (LOW RISK): All data were collected directly from the subjects.<br>☐ No (HIGH RISK): In some instances, data were collected from a proxy

6. Was an acceptable case definition used in the study?	□ Yes (LOW RISK): An acceptable case definition was used.<br>□ No (HIGH RISK): An acceptable case definition was NOT used.
7. Was the study instrument that measured the parameter of interest (e.g. prevalence of low back pain) shown to have reliability and validity (if necessary)?	□ Yes (LOW RISK): The study instrument had been shown to have reliability and validity (if this was necessary), e.g. test-retest, piloting, validation in a previous study, etc.<br>□ No (HIGH RISK): The study instrument had NOT been shown to have reliability or validity (if this was necessary).
8. Was the same mode of data collection used for all subjects?	□ Yes (LOW RISK): The same mode of data collection was used for all subjects.<br>□ No (HIGH RISK): The same mode of data collection was NOT used for all subjects.
9. Was the length of the shortest prevalence period for the parameter of interest appropriate?	□Yes (LOW RISK): The shortest prevalence period for the parameter of interest was appropriate (e.g. point prevalence, one-week prevalence, one-year prevalence).<br>□ No (HIGH RISK): The shortest prevalence period for the parameter of interest was not appropriate (e.g. lifetime prevalence)
10. Were the numerator(s) and denominator(s) for the parameter of interest appropriate?	□Yes (LOW RISK): The paper presented appropriate numerator(s) AND denominator(s) for the parameter of interest (e.g. the prevalence of low back pain).<br>□ No (HIGH RISK): The paper did present numerator(s) AND denominator(s) for the parameter of interest but one or more of these were inappropriate

Supplemental Reference

51. Garon EB, Aerts J, Kim JS*, et al.* Safety of pemetrexed plus platinum in combination with pembrolizumab for metastatic nonsquamous non-small cell lung cancer: A post hoc analysis of KEYNOTE-189. Lung Cancer 2021;155:53-60

52. Goldberg SB, Schalper KA, Gettinger SN*, et al.* Pembrolizumab for management of patients with NSCLC and brain metastases: long-term results and biomarker analysis from a non-randomised, open-label, phase 2 trial. Lancet Oncol 2020;21(5):655-663

53. Middleton G, Brock K, Savage J*, et al.* Pembrolizumab in patients with non-small-cell lung cancer of performance status 2 (PePS2): a single arm, phase 2 trial. Lancet Respir Med 2020;8(9):895-904

54. Powles T, van der Heijden MS, Castellano D*, et al.* Durvalumab alone and durvalumab plus tremelimumab versus chemotherapy in previously untreated patients with unresectable, locally advanced or metastatic urothelial carcinoma (DANUBE): a randomised, open-label, multicentre, phase 3 trial. Lancet Oncol 2020;21(12):1574-1588

55. Malhotra J, Nikolinakos P, Leal T*, et al.* A Phase 1-2 Study of Rovalpituzumab Tesirine in Combination With Nivolumab Plus or Minus Ipilimumab in Patients With Previously Treated Extensive-Stage SCLC. J Thorac Oncol 2021;16(9):1559-1569

56. Trevisani F, Di Marco F, Raggi D*, et al.* Renal function outcomes in patients with muscle-invasive bladder cancer treated with neoadjuvant pembrolizumab and radical cystectomy in the PURE-01 study. Int J Cancer 2021;149(1):186-190

57. Yu EY, Petrylak DP, O'Donnell PH*, et al.* Enfortumab vedotin after PD-1 or PD-L1 inhibitors in cisplatin-ineligible patients with advanced urothelial carcinoma (EV-201): a multicentre, single-arm, phase 2 trial. Lancet Oncol 2021;22(6):872-882

58. Altman D, Weight RM, Shah MR*, et al.* Thyroid-related laboratory abnormalities to predict treatment-limiting adverse events in melanoma patients treated with immune checkpoint blockade. American Society of Clinical Oncology; 2016.

59. Antonia SJ, Balmanoukian A, Brahmer J*, et al.* Clinical Activity, Tolerability, and Long-Term Follow-Up of Durvalumab in Patients With Advanced NSCLC. J Thorac Oncol 2019;14(10):1794-1806

60. Antonia SJ, Borghaei H, Ramalingam SS*, et al.* Four-year survival with nivolumab in patients with previously treated advanced non-small-cell lung cancer: a pooled analysis. Lancet Oncol 2019;20(10):1395-1408

61. Armand P, Lesokhin A, Borrello I*, et al.* A phase 1b study of dual PD-1 and CTLA-4 or KIR blockade in patients with relapsed/refractory lymphoid malignancies. Leukemia 2021;35(3):777-786

62. Balar AV, Castellano D, O'Donnell PH*, et al.* First-line pembrolizumab in cisplatin-

ineligible patients with locally advanced and unresectable or metastatic urothelial cancer (KEYNOTE-052): a multicentre, single-arm, phase 2 study. Lancet Oncol 2017;18(11):1483-1492
63. Basak EA, Vermeer NS, de Joode K*, et al.* Associations between patient and disease characteristics and severe adverse events during immune checkpoint inhibitor treatment: An observational study. Eur J Cancer 2022;174:113-120
64. Barlesi F, Vansteenkiste J, Spigel D*, et al.* Avelumab versus docetaxel in patients with platinum-treated advanced non-small-cell lung cancer (JAVELIN Lung 200): an open-label, randomised, phase 3 study. Lancet Oncol 2018;19(11):1468-1479
65. Bellmunt J, Hussain M, Gschwend JE*, et al.* Adjuvant atezolizumab versus observation in muscle-invasive urothelial carcinoma (IMvigor010): a multicentre, open-label, randomised, phase 3 trial. Lancet Oncol 2021;22(4):525-537
66. Calabro L, Morra A, Giannarelli D*, et al.* Tremelimumab combined with durvalumab in patients with mesothelioma (NIBIT-MESO-1): an open-label, non-randomised, phase 2 study. Lancet Respir Med 2018;6(6):451-460
67. Le Cesne A, Marec-Berard P, Blay JY*, et al.* Programmed cell death 1 (PD-1) targeting in patients with advanced osteosarcomas: results from the PEMBROSARC study. Eur J Cancer 2019;119:151-157
68. Chau I, Penel N, Soriano AO*, et al.* Ramucirumab in Combination with Pembrolizumab in Treatment-Naive Advanced Gastric or GEJ Adenocarcinoma: Safety and Antitumor Activity from the Phase 1a/b JVDF Trial. Cancers (Basel) 2020;12(10)
69. Desai J, Fong P, Moreno V*, et al.* A Phase 1/2 study of the PD-L1 inhibitor, BGB-A333, alone and in combination with the PD-1 inhibitor, tislelizumab, in patients with advanced solid tumours. Br J Cancer 2023;128(8):1418-1428
70. Feun LG, Li YY, Wu C*, et al.* Phase 2 study of pembrolizumab and circulating biomarkers to predict anticancer response in advanced, unresectable hepatocellular carcinoma. Cancer 2019;125(20):3603-3614
71. Guven DC, Ozbek DA, Sahin TK*, et al.* The incidence and risk factors for acute kidney injury in patients treated with immune checkpoint inhibitors. Anticancer Drugs 2023;34(6):783-790
72. Hellmann MD, Rizvi NA, Goldman JW*, et al.* Nivolumab plus ipilimumab as first-line treatment for advanced non-small-cell lung cancer (CheckMate 012): results of an open-label, phase 1, multicohort study. Lancet Oncol 2017;18(1):31-41
73. Hoffman-Censits J, Pal S, Kaiser C*, et al.* Atezolizumab in patients with renal insufficiency and mixed variant histology: analyses from an expanded access program in platinum-treated locally advanced or metastatic urothelial carcinoma. J Immunother Cancer 2020;8(2)
74. Kanbay M, Yildiz AB, Siriopol D*, et al.* Immune checkpoints inhibitors and its link

to acute kidney injury and renal prognosis. Int Urol Nephrol 2023;55(4):1025-1032
75. Lee EK, Xiong N, Cheng SC, *et al.* Combined pembrolizumab and pegylated liposomal doxorubicin in platinum resistant ovarian cancer: A phase 2 clinical trial. Gynecol Oncol 2020;159(1):72-78
76. Long GV, Dummer R, Hamid O, *et al.* Epacadostat plus pembrolizumab versus placebo plus pembrolizumab in patients with unresectable or metastatic melanoma (ECHO-301/KEYNOTE-252): a phase 3, randomised, double-blind study. Lancet Oncol 2019;20(8):1083-1097
77. Manohar S, Goksu BNB, Finnes HD, *et al.* Prevalence of AKI in PD-1 inhibitors: A single center experience. Journal of the American Society of Nephrology 2018;29:1034
78. Mateos MV, Orlowski RZ, Ocio EM, *et al.* Pembrolizumab combined with lenalidomide and low-dose dexamethasone for relapsed or refractory multiple myeloma: phase I KEYNOTE-023 study. Br J Haematol 2019;186(5):e117-e121
79. Moreau P, Ghori R, Farooqui M, *et al.* Pembrolizumab combined with carfilzomib and low-dose dexamethasone for relapsed or refractory multiple myeloma: Cohort 2 of the phase I KEYNOTE-023 study. Br J Haematol 2021;194(1):e48-e51
80. Raghav KP, Stephen B, Karp DD, *et al.* Efficacy of pembrolizumab in patients with advanced cancer of unknown primary (CUP): a phase 2 non-randomized clinical trial. J Immunother Cancer 2022;10(5)
81. Rao S, Anandappa G, Capdevila J, *et al.* A phase II study of retifanlimab (INCMGA00012) in patients with squamous carcinoma of the anal canal who have progressed following platinum-based chemotherapy (POD1UM-202). ESMO Open 2022;7(4):100529
82. Rizvi NA, Hellmann MD, Brahmer JR, *et al.* Nivolumab in Combination With Platinum-Based Doublet Chemotherapy for First-Line Treatment of Advanced Non-Small-Cell Lung Cancer. J Clin Oncol 2016;34(25):2969-2979
83. Rodrigues M, Vanoni G, Loap P, *et al.* Nivolumab plus chemoradiotherapy in locally-advanced cervical cancer: the NICOL phase 1 trial. Nat Commun 2023;14(1):3698
84. Rose TL, Harrison MR, Deal AM, *et al.* Phase II Study of Gemcitabine and Split-Dose Cisplatin Plus Pembrolizumab as Neoadjuvant Therapy Before Radical Cystectomy in Patients With Muscle-Invasive Bladder Cancer. J Clin Oncol 2021;39(28):3140-3148
85. Seydel F, Delecluse S, Zeier M, *et al.* Efficacy and Safety of Checkpoint Inhibitor Treatment in Patients with Advanced Renal or Urothelial Cell Carcinoma and Concomitant Chronic Kidney Disease: A Retrospective Cohort Study. Cancers (Basel) 2021;13(7)

86. Sezer A, Kilickap S, Gumus M*, et al.* Cemiplimab monotherapy for first-line treatment of advanced non-small-cell lung cancer with PD-L1 of at least 50%: a multicentre, open-label, global, phase 3, randomised, controlled trial. Lancet 2021;397(10274):592-604
87. Sharma P, Callahan MK, Bono P*, et al.* Nivolumab monotherapy in recurrent metastatic urothelial carcinoma (CheckMate 032): a multicentre, open-label, two-stage, multi-arm, phase 1/2 trial. Lancet Oncol 2016;17(11):1590-1598
88. Sonpavde GP, Maughan BL, McGregor BA*, et al.* Phase II trial of CV301 vaccine combined with atezolizumab in advanced urothelial carcinoma. Cancer Immunol Immunother 2023;72(3):775-782
89. Wolchok JD, Kluger H, Callahan MK*, et al.* Nivolumab plus ipilimumab in advanced melanoma. N Engl J Med 2013;369(2):122-133
90. Winer EP, Lipatov O, Im SA*, et al.* Pembrolizumab versus investigator-choice chemotherapy for metastatic triple-negative breast cancer (KEYNOTE-119): a randomised, open-label, phase 3 trial. Lancet Oncol 2021;22(4):499-511
91. Wise-Draper TM, Gulati S, Palackdharry S*, et al.* Phase II Clinical Trial of Neoadjuvant and Adjuvant Pembrolizumab in Resectable Local-Regionally Advanced Head and Neck Squamous Cell Carcinoma. Clin Cancer Res 2022;28(7):1345-1352
92. Yu X, Wu R, Ji Y*, et al.* Identifying Patients at Risk of Acute Kidney Injury among Patients Receiving Immune Checkpoint Inhibitors: A Machine Learning Approach. Diagnostics (Basel) 2022;12(12)
93. Zinzani PL, Santoro A, Gritti G*, et al.* Nivolumab Combined With Brentuximab Vedotin for Relapsed/Refractory Primary Mediastinal Large B-Cell Lymphoma: Efficacy and Safety From the Phase II CheckMate 436 Study. J Clin Oncol 2019;37(33):3081-3089
94. Tanizaki J, Yonemori K, Akiyoshi K*, et al.* Open-label phase II study of the efficacy of nivolumab for cancer of unknown primary. Ann Oncol 2022;33(2):216-226
95. Balanchivadze N, Nasser Z, Shahid M*, et al.* 1317P Renal toxicity in black patients with non-squamous non-small cell lung cancer treated with combination platinum-pemetrexed-pembrolizumab therapy. Annals of Oncology 2021;32:S1013
96. Lumlertgul N, Vassallo P, Tydeman F*, et al.* Acute kidney injury in patients receiving immune checkpoint inhibitors: a retrospective real-world study. Eur J Cancer 2023;191:112967
97. Lou Q, Gong J, Ye B*, et al.* Acute kidney injury in patients with cancer receiving anti-PD-1/PD-L1 antibodies: incidence, risk factors, and prognosis. Ren Fail 2023;45(1):2238823
98. Dirix LY, Takacs I, Jerusalem G*, et al.* Avelumab, an anti-PD-L1 antibody, in patients with locally advanced or metastatic breast cancer: a phase 1b JAVELIN Solid

Tumor study. Breast Cancer Res Treat 2018;167(3):671-686
99. Stratigos AJ, Sekulic A, Peris K*, et al.* Cemiplimab in locally advanced basal cell carcinoma after hedgehog inhibitor therapy: an open-label, multi-centre, single-arm, phase 2 trial. Lancet Oncol 2021;22(6):848-857
100. Dang E, Vallee A, Lepage-Seydoux C*, et al.* Clinical Benefit of Pembrolizumab in Advanced Urothelial Cancer Patients in Real-Life Setting: An Efficacy and Safety Monocentric Study. Curr Oncol 2022;29(2):945-955
101. Gulati S, Crist M, Riaz MK*, et al.* Durvalumab plus Cetuximab in Patients with Recurrent or Metastatic Head and Neck Squamous Cell Carcinoma: An Open-label, Nonrandomized, Phase II Clinical Trial. Clin Cancer Res 2023;29(10):1906-1915
102. Rosenberg JE, Park SH, Kozlov V*, et al.* Durvalumab Plus Olaparib in Previously Untreated, Platinum-Ineligible Patients With Metastatic Urothelial Carcinoma: A Multicenter, Randomized, Phase II Trial (BAYOU). J Clin Oncol 2023;41(1):43-53
103. Tanaka T, Hatakeyama S, Numakura K*, et al.* Efficacy and safety of first-line nivolumab plus ipilimumab in patients with metastatic renal cell carcinoma: A multicenter retrospective study. Int J Urol 2020;27(12):1095-1100
104. Fedorova LV, Lepik KV, Volkov NP*, et al.* Efficacy and safety of nivolumab combined with brentuximab vedotin after nivolumab monotherapy failure in patients with relapsed and refractory classic Hodgkin lymphoma. Int J Clin Oncol 2022;27(3):626-632
105. Martin C, Lupinacci L, Perazzo F*, et al.* Efficacy and Safety of Nivolumab in Previously Treated Patients With Non-Small-cell Lung Cancer: Real World Experience in Argentina. Clin Lung Cancer 2020;21(5):e380-e387
106. Paz-Ares L, Ciuleanu TE, Cobo M*, et al.* First-line nivolumab plus ipilimumab combined with two cycles of chemotherapy in patients with non-small-cell lung cancer (CheckMate 9LA): an international, randomised, open-label, phase 3 trial. Lancet Oncol 2021;22(2):198-211
107. Dummer R, Corrie P, Gutzmer R*, et al.* First-Line, Fixed-Duration Nivolumab Plus Ipilimumab Followed by Nivolumab in Clinically Diverse Patient Populations With Unresectable Stage III or IV Melanoma: CheckMate 401. J Clin Oncol 2023;41(23):3917-3929
108. Heppt MV, Eigentler TK, Kahler KC*, et al.* Immune checkpoint blockade with concurrent electrochemotherapy in advanced melanoma: a retrospective multicenter analysis. Cancer Immunol Immunother 2016;65(8):951-959
109. Knox A, Cloney T, Janssen H*, et al.* Immune-related acute kidney injury in Australian non-small cell lung cancer patients: Real-world results. Lung Cancer 2023;184:107325
110. Egelston CA, Guo W, Yost SE*, et al.* Immunogenicity and efficacy of

pembrolizumab and doxorubicin in a phase I trial for patients with metastatic triple-negative breast cancer. Cancer Immunol Immunother 2023;72(9):3013-3027
111. Ascierto PA, Del Vecchio M, Robert C*, et al.* Ipilimumab 10 mg/kg versus ipilimumab 3 mg/kg in patients with unresectable or metastatic melanoma: a randomised, double-blind, multicentre, phase 3 trial. Lancet Oncol 2017;18(5):611-622
112. Garcia P, Stedman MR, Merlo S*, et al.* Kidney function while on immune checkpoint inhibitors: Trends in incidence of acute kidney injury, and its causes and outcomes. Journal of Onco-Nephrology 2023:23993693231161875
113. Leon CA. Nephrotoxicity due to immune checkpoint inhibitors in rural oncology. American Society of Clinical Oncology; 2020.
114. Reiss KA, Mick R, Teitelbaum U*, et al.* Niraparib plus nivolumab or niraparib plus ipilimumab in patients with platinum-sensitive advanced pancreatic cancer: a randomised, phase 1b/2 trial. Lancet Oncol 2022;23(8):1009-1020
115. Overman MJ, McDermott R, Leach JL*, et al.* Nivolumab in patients with metastatic DNA mismatch repair-deficient or microsatellite instability-high colorectal cancer (CheckMate 142): an open-label, multicentre, phase 2 study. Lancet Oncol 2017;18(9):1182-1191
116. Motzer RJ, Rini BI, McDermott DF*, et al.* Nivolumab plus ipilimumab versus sunitinib in first-line treatment for advanced renal cell carcinoma: extended follow-up of efficacy and safety results from a randomised, controlled, phase 3 trial. Lancet Oncol 2019;20(10):1370-1385
117. Gettinger SN, Redman MW, Bazhenova L*, et al.* Nivolumab Plus Ipilimumab vs Nivolumab for Previously Treated Patients With Stage IV Squamous Cell Lung Cancer: The Lung-MAP S1400I Phase 3 Randomized Clinical Trial. JAMA Oncol 2021;7(9):1368-1377
118. Dizman N, Meza L, Bergerot P*, et al.* Nivolumab plus ipilimumab with or without live bacterial supplementation in metastatic renal cell carcinoma: a randomized phase 1 trial. Nat Med 2022;28(4):704-712
119. Vano YA, Elaidi R, Bennamoun M*, et al.* Nivolumab, nivolumab-ipilimumab, and VEGFR-tyrosine kinase inhibitors as first-line treatment for metastatic clear-cell renal cell carcinoma (BIONIKK): a biomarker-driven, open-label, non-comparative, randomised, phase 2 trial. Lancet Oncol 2022;23(5):612-624
120. Albiges L, Gurney H, Atduev V*, et al.* Pembrolizumab plus lenvatinib as first-line therapy for advanced non-clear-cell renal cell carcinoma (KEYNOTE-B61): a single-arm, multicentre, phase 2 trial. Lancet Oncol 2023;24(8):881-891
121. Kuruvilla J, Ramchandren R, Santoro A*, et al.* Pembrolizumab versus brentuximab vedotin in relapsed or refractory classical Hodgkin lymphoma

(KEYNOTE-204): an interim analysis of a multicentre, randomised, open-label, phase 3 study. Lancet Oncol 2021;22(4):512-524
122. Powles T, Tomczak P, Park SH*, et al.* Pembrolizumab versus placebo as post-nephrectomy adjuvant therapy for clear cell renal cell carcinoma (KEYNOTE-564): 30-month follow-up analysis of a multicentre, randomised, double-blind, placebo-controlled, phase 3 trial. Lancet Oncol 2022;23(9):1133-1144
123. D'Souza A, Hari P, Pasquini M*, et al.* A Phase 2 Study of Pembrolizumab during Lymphodepletion after Autologous Hematopoietic Cell Transplantation for Multiple Myeloma. Biol Blood Marrow Transplant 2019;25(8):1492-1497
124. Tsung I, Green E, Palmbos P*, et al.* A Phase 2 Trial of Nab-paclitaxel in Combination With Anti-PD1 Therapy in Advanced Urothelial Cancer. J Urol 2023;209(1):121-130
125. Tuscano JM, Maverakis E, Groshen S*, et al.* A Phase I Study of the Combination of Rituximab and Ipilimumab in Patients with Relapsed/Refractory B-Cell Lymphoma. Clin Cancer Res 2019;25(23):7004-7013
126. Li H, Sahu KK, Brundage J*, et al.* Phase I Trial of Combination Therapy With Avelumab and Cabozantinib in Patients With Newly Diagnosed Metastatic Clear Cell Renal Cell Carcinoma. Oncologist 2023;28(8):737-e693
127. Taylor MH, Lee CH, Makker V*, et al.* Phase IB/II Trial of Lenvatinib Plus Pembrolizumab in Patients With Advanced Renal Cell Carcinoma, Endometrial Cancer, and Other Selected Advanced Solid Tumors. J Clin Oncol 2020;38(11):1154-1163
128. Patel SP, Othus M, Chae YK*, et al.* A Phase II Basket Trial of Dual Anti-CTLA-4 and Anti-PD-1 Blockade in Rare Tumors (DART SWOG 1609) in Patients with Nonpancreatic Neuroendocrine Tumors. Clin Cancer Res 2020;26(10):2290-2296
129. Oh CR, Kim JE, Hong YS*, et al.* Phase II study of durvalumab monotherapy in patients with previously treated microsatellite instability-high/mismatch repair-deficient or POLE-mutated metastatic or unresectable colorectal cancer. Int J Cancer 2022;150(12):2038-2045
130. Brastianos PK, Strickland MR, Lee EQ*, et al.* Phase II study of ipilimumab and nivolumab in leptomeningeal carcinomatosis. Nat Commun 2021;12(1):5954
131. Yam C, Mittendorf EA, Garber HR*, et al.* A phase II study of neoadjuvant atezolizumab and nab-paclitaxel in patients with anthracycline-resistant early-stage triple-negative breast cancer. Breast Cancer Res Treat 2023;199(3):457-469
132. Carlo MI, Attalla K, Mazaheri Y*, et al.* Phase II Study of Neoadjuvant Nivolumab in Patients with Locally Advanced Clear Cell Renal Cell Carcinoma Undergoing Nephrectomy. Eur Urol 2022;81(6):570-573
133. Shah AN, Flaum L, Helenowski I*, et al.* Phase II study of pembrolizumab and

capecitabine for triple negative and hormone receptor-positive, HER2-negative endocrine-refractory metastatic breast cancer. J Immunother Cancer 2020;8(1)
134. Sternberg CN, Loriot Y, James N*, et al.* Primary Results from SAUL, a Multinational Single-arm Safety Study of Atezolizumab Therapy for Locally Advanced or Metastatic Urothelial or Nonurothelial Carcinoma of the Urinary Tract. Eur Urol 2019;76(1):73-81
135. Herbst RS, Arkenau HT, Santana-Davila R*, et al.* Ramucirumab plus pembrolizumab in patients with previously treated advanced non-small-cell lung cancer, gastro-oesophageal cancer, or urothelial carcinomas (JVDF): a multicohort, non-randomised, open-label, phase 1a/b trial. Lancet Oncol 2019;20(8):1109-1123
136. Baggi A, Quaglino P, Rubatto M*, et al.* Real world data of cemiplimab in locally advanced and metastatic cutaneous squamous cell carcinoma. Eur J Cancer 2021;157:250-258
137. Grimm MO, Grunwald V, Muller-Huesmann H*, et al.* Real-World Data on the Use of Nivolumab Monotherapy in the Treatment of Advanced Renal Cell Carcinoma after Prior Therapy: Interim Results from the Noninterventional NORA Study. Eur Urol Focus 2022;8(5):1289-1299
138. Taylor MH, Betts CB, Maloney L*, et al.* Safety and Efficacy of Pembrolizumab in Combination with Acalabrutinib in Advanced Head and Neck Squamous Cell Carcinoma: Phase 2 Proof-of-Concept Study. Clin Cancer Res 2022;28(5):903-914
139. Irwin CT, Panic J, Lodhi FA*, et al.* A single-center cohort study of nephrotoxicity due to immune checkpoint inhibitors. Journal of the American Society of Nephrology 2020;31:661-662
140. Mushtaq S, Kerr DA, Mirza A-S*, et al.* A single-institution study of renal outcomes in patients receiving checkpoint inhibitors. American Society of Clinical Oncology; 2018.
141. Moledina DG, Eadon MT, Calderon F*, et al.* Development and external validation of a diagnostic model for biopsy-proven acute interstitial nephritis using electronic health record data. Nephrol Dial Transplant 2022;37(11):2214-2222
142. Christiansen CF, Johansen MB, Langeberg WJ*, et al.* Incidence of acute kidney injury in cancer patients: a Danish population-based cohort study. Eur J Intern Med 2011;22(4):399-406
143. Jin J, Wang Y, Shen Q*, et al.* Acute kidney injury in cancer patients: A nationwide survey in China. Sci Rep 2019;9(1):3540
144. Kitchlu A, McArthur E, Amir E*, et al.* Acute Kidney Injury in Patients Receiving Systemic Treatment for Cancer: A Population-Based Cohort Study. J Natl Cancer Inst 2019;111(7):727-736
145. Carlos CA, Hsu RK. Acute Kidney Injury with Immune Checkpoint Inhibitors: A

Push beyond Case Reports. Clin J Am Soc Nephrol 2019;14(12):1679-1681
146. Jaworska K, Ratajczak J, Huang L*, et al.* Both PD-1 ligands protect the kidney from ischemia reperfusion injury. J Immunol 2015;194(1):325-333
147. Menke J, Lucas JA, Zeller GC*, et al.* Programmed death 1 ligand (PD-L) 1 and PD-L2 limit autoimmune kidney disease: distinct roles. J Immunol 2007;179(11):7466-7477
148. Singh S, Clemente LC, Parra ER*, et al.* Urinary T cells are detected in patients with immune checkpoint inhibitor-associated immune nephritis that are clonotypically identical to kidney T cell infiltrates. Oncoimmunology 2022;11(1):2124678
